# The role of WNT10B in physiology and disease: A 10-year update

**DOI:** 10.3389/fcell.2023.1120365

**Published:** 2023-02-06

**Authors:** Rachel S. Perkins, Rishika Singh, Amy N. Abell, Susan A. Krum, Gustavo A. Miranda-Carboni

**Affiliations:** ^1^ Department of Orthopaedic Surgery and Biomedical Engineering, University of Tennessee Health Science Center, Memphis, TN, United States; ^2^ College of Medicine, University of Tennessee Health Science Center, Memphis, TN, United States; ^3^ Department of Biological Sciences, University of Memphis, Memphis, TN, United States; ^4^ Center for Cancer Research, University of Tennessee Health Science Center, Memphis, TN, United States; ^5^ Department of Medicine, Division of Hematology and Oncology, College of Medicine, University of Tennessee Health Science Center, Memphis, TN, United States

**Keywords:** WNT10B, Wnt, Wnt signaling, bone, disease, cancer, development

## Abstract

WNT10B, a member of the WNT family of secreted glycoproteins, activates the WNT/β-catenin signaling cascade to control proliferation, stemness, pluripotency, and cell fate decisions. WNT10B plays roles in many tissues, including bone, adipocytes, skin, hair, muscle, placenta, and the immune system. Aberrant WNT10B signaling leads to several diseases, such as osteoporosis, obesity, split-hand/foot malformation (SHFM), fibrosis, dental anomalies, and cancer. We reviewed WNT10B a decade ago, and here we provide a comprehensive update to the field. Novel research on WNT10B has expanded to many more tissues and diseases. *WNT10B* polymorphisms and mutations correlate with many phenotypes, including bone mineral density, obesity, pig litter size, dog elbow dysplasia, and cow body size. In addition, the field has focused on the regulation of *WNT10B* using upstream mediators, such as microRNAs (miRNAs) and long non-coding RNAs (lncRNAs). We also discussed the therapeutic implications of WNT10B regulation. In summary, research conducted during 2012–2022 revealed several new, diverse functions in the role of WNT10B in physiology and disease.

## 1 Introduction

WNT10B was first discovered by the Leder group in the mammary gland of mice in 1995 (Lee et al., 1995). Since then, WNT10B has been shown to play important roles in many tissue types in normal development (including bone, adipocytes, teeth, skin, hair, immune system, muscle, placenta, and heart) and diseases [including cancer, obesity, osteoporosis, and split-hand/foot malformation (SHFM)]. WNT10B was reviewed a decade ago (Wend et al., 2012). Here, we provide an update on the current understanding of WNT10B in normal development and disease from 2012 to 2022.

## 2 WNT10B signaling

**FIGURE 1:**
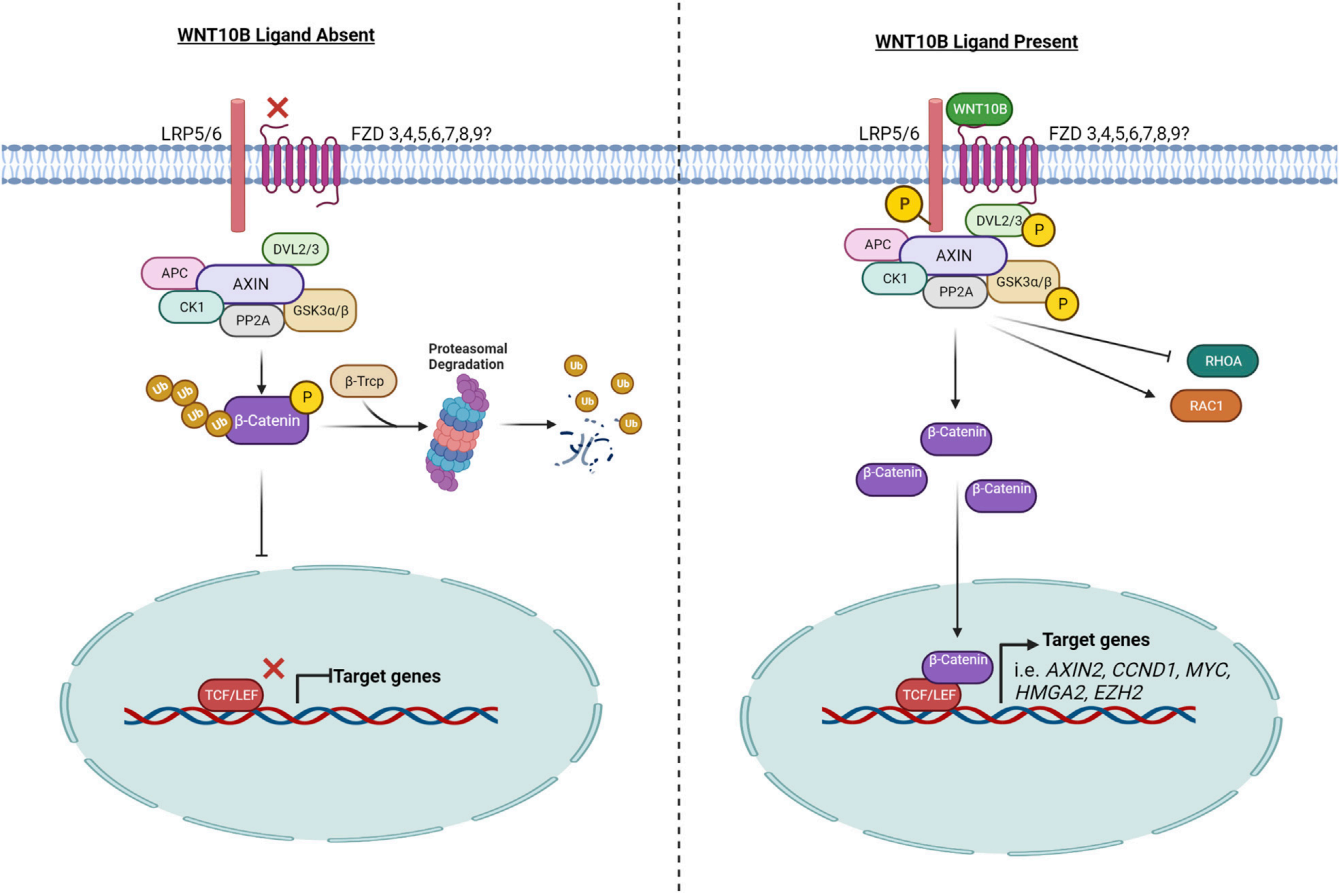
WNT10B signaling pathway. The left side of the figure represents the signaling pathway when WNT10B is absent and the signaling pathway is off. The right side of the figure models the signaling pathway when WNT10B is present, following from receptor binding to target gene activation. P, phosphorylation; Ub, ubiquitination. Created with BioRender.com.

WNT ligands (e.g., WNT1, WNT3A, and WNT10B) can activate signal transduction in a β-catenin-dependent manner, referred to as “canonical” WNT/β-catenin signaling, and/or in a β-catenin-independent manner (e.g., WNT5A, WNT5B, and WNT7A), referred to as “non-canonical” WNT/β-catenin signaling. WNT10B is considered a “canonical”/β-catenin-dependent WNT ligand. Many WNT signaling reviews have summarized the signaling pathway with data combined from a variety of species and WNT ligands (MacDonald et al., 2009; Rim et al., 2022). Here, we will specifically summarize what is known for WNT10B (Figure 1).

Canonical WNTs bind to LRP5 and/or LRP6 and a frizzled (FZD) co-receptor to initiate signaling. WNT10B has been shown to interact with both LRP5 and LRP6 in different tissue-specific contexts. In the prostate cancer cell line PC3, WNT10B was shown by proximal ligation assay (PLA) to co-localize with FZD6 and LRP6 and then induce nuclear β-catenin in 5 min (Neuhaus et al., 2021). In osteoblasts, WNT10B upregulates the transcription of fatty acid metabolism genes *via* LRP5 (but not LRP6) (Frey et al., 2015). In addition, WNT10B expression is often correlated with LRP5 expression (Belaya et al., 2018; Li Z. et al., 2021). LRP5 and LRP6 are both required for WNT signaling reporter assay activity in response to WNT10B in mouse embryonic fibroblasts (Goel et al., 2012).

A molecular docking simulation study was performed to predict the binding affinity of WNTs with various human FZDs, and it predicted that human WNT10B has the highest binding affinity with human FZD3, 4, 5, 6, 7, 8, and 9 (Agostino et al., 2017). Tissue-specific receptor and co-receptor expression may determine ligand binding. Specifically in acute myeloid leukemia (AML), WNT10B signals through FZD4 and FZD5, while in T-cell acute lymphoblastic leukemia, WNT10B signals through FZD6 (Lazzaroni et al., 2016; Cassaro et al., 2021). WNT10B also interacted with FZD6 in a T-cell acute lymphoblastic leukemia cell line and HEK293T cells, as demonstrated by immunoprecipitation and PLA (Lu et al., 2019; Cassaro et al., 2021). In BeWo choriocarcinoma cells, WNT10B signaling requires FZD7 for migration but not the other expressed FZD family members (FZD1, FZD3, FZD5, and FZD10) (Wang et al., 2019).

The signaling output of the canonical WNT pathway is determined by the level of cytosolic β-catenin, which is under the strict control of the “destruction complex.” The core destruction complex is composed of AXIN, APC, and two constitutively active kinases [casein kinase 1 (CK1) and glycogen synthase kinase 3 (GSK3α/β)], which associate with β-catenin and promote its polyubiquitination by phosphorylating the degron motif of β-catenin (Stamos and Weis, 2013). Phosphorylated β-CATENIN is presented to the proteasome through its interaction with the F-box/WD-repeat containing E3-ligase protein β-TrCP, an adaptor protein that forms a complex with the SKP1/Cullin machinery, to facilitate assembly of K48-linked polyubiquitin chains on β-catenin, leading to it proteasome-dependent degradation (Kitagawa et al., 1999).

WNT ligands, including WNT10B (Lee et al., 2013), lead to the phosphorylation of GSK3β, which inhibits the destruction complex. Therefore, WNT10B stabilizes and activates β-catenin, as demonstrated by analysis of β-catenin (Abiola et al., 2009; Lei et al., 2015). Increased cytoplasmic β-catenin then translocates into the nucleus where it binds to members of the T cell factor/lymphoid enhancer factor (TCF/LEF) transcription factor family to drive transcription of WNT/β-catenin target genes, such as MYC, CCND1, and AXIN2. WNT10B activates the TOPFLASH reporter, a canonical WNT signaling reporter with TCF/LEF binding sites, which has been widely used as a readout of activated WNT10B signaling (Christodoulides et al., 2006; Suthon et al., 2022a).

WNT binding to FZD helps recruit DVL to the plasma membrane. Recruitment of DVL to the membrane provides a platform for AXIN and GSK3β to bind and phosphorylate LRP5/6, thereby preventing constitutive degradation of β-catenin (Sharma et al., 2018). In several studies, WNT10B was shown to induce DVL2 and/or DVL3 phosphorylation (Chen et al., 2008; Golestaneh et al., 2009; Lee and Heur, 2015).

WNT10B signals through RAC1, which is part of the canonical and non-canonical pathways. WNT10B can signal *via* FZD7 to activate RAC1 in placental extravillous trophoblasts to mediate migration (Wang et al., 2019). In corneal endothelial cells, WNT10B treatment caused an activation of RAC1 and an association of RAC1 and β-catenin in the nucleus. In contrast, WNT10B inhibited RHOA (a member of the same GTPase family as RAC1) activity (Lee and Heur, 2015).

## 3 WNT10B and the mammary gland

WNT10B, which was first identified in the mammary gland, plays a very fundamental role in mammary gland development. It is the earliest (E11.5) expressed WNT ligand in the mammary anlagen and is a characteristic of the definitive mammary line (Veltmaat et al., 2004). However, research in the past decade has not focused on WNT10B in the normal mammary gland but on its role in breast cancer and other tissues (discussed in following sections).

## 4 WNT10B and bone

WNT signaling is an important regulator of bone. Human mutations in LRP5 and SOST (sclerostin) alter WNT signaling and cause osteoporosis-pseudoglioma syndrome (OPPG) and van Buchem disease. Romosozumab (EVENITY), an antibody to sclerostin which activates WNT signaling, is an FDA-approved therapy for osteoporosis. In mice, knockouts of LRP5, LRP6, β-catenin, and many WNT ligands each have skeletal phenotypes (Maupin et al., 2013). In the previous decade, WNT10B-knockout mice and transgenic mice demonstrated the importance of WNT10B in bone and bone marrow-derived mesenchymal stem cells (Bennett et al., 2005; Bennett et al., 2007; Stevens et al., 2010). More recently, studies have focused on the regulation of *WNT10B* in bone and the therapeutic applications of WNT10B in bone.

Analysis of *WNT10B* single nucleotide polymorphisms (SNPs) has also supported a role for WNT10B in bone. A 2009 study demonstrated an association of two SNPs (rs1051886 and rs3741627) in the *WNT10B* gene with high hip bone-mass density and increased bone size in Afro-Caribbean men (Zmuda et al., 2009). A study on Danish men also showed a correlation of SNP rs10875902 (which is in high linkage disequilibrium with rs1051886) with bone mineral density (Van Camp et al., 2013). However, *WNT10B*-targeted studies in Spanish (Perez-Castrillon et al., 2009) and Chinese (Zheng et al., 2016) populations, as well as large GWAS (Estrada et al., 2012; Liu et al., 2020c), did not identify any correlations between *WNT10B* SNPs and bone properties.

WNT10B affects mesenchymal stem cells (MSCs), osteoblasts, osteoclasts, and T cells, which contribute to bone homeostasis. The effects of WNT10B from T cells affecting the bone are discussed later in the review (see Section 5: WNT10B and the immune system).

MSCs differentiate into osteoblasts, adipocytes, chondrocytes, myocytes, and other mesenchymal tissues. Osteoblasts and adipocytes have an inverse relationship; factors that increase osteoblastogenesis tend to decrease adipogenesis and *vice versa*. WNT10B is considered a master negative regulator of adipocyte differentiation (see Section 8). Thus, WNT10B is often used as a marker of increased osteoblastogenesis, decreased adipogenesis, or both (Liu et al., 2016; Matsushita et al., 2016; Yi et al., 2019; Palmieri et al., 2021), for example in bone marrow adipocytes (Georgiou et al., 2015; Lee et al., 2019).

An interesting model to study bone biology is the scales of goldfish. Teleost scales are formed by intramembranous ossification and comprise osteoblasts and osteoclasts. Tazaki et al. (2018) detected *WNT10B* by qPCR and *in situ* hybridization in multinucleated osteoclasts in fish scales. Furthermore, the inhibition of WNT10B with an anti-WNT10B antibody increased RANKL expression, increasing osteoclast differentiation (Tazaki et al., 2018), thereby supporting the role of WNT10B in increasing bone mineral density.

The majority of studies on WNT10B in the bone have focused on osteoblasts, and WNT10B is also expressed in osteoclasts. It is hypothesized that the WNT10B produced from osteoclasts links the coupling of osteoclasts and osteoblasts in the resorption cycle, inducing paracrine signaling to osteoblasts from the osteoclasts (Ota et al., 2013; Zheng et al., 2019). A loss-of-function mutation of *c-kit* in mice led to an increase in osteoclast expression of WNT10B and an increase in osteoblast differentiation and mineralization that could be blocked with a WNT10B neutralizing antibody or addition of recombinant DKK1 (Lotinun and Krishnamra, 2016). In the studies on WNT10B expression in osteoclasts, no direct effect of WNT10B on osteoclasts is shown, only the effect on osteoblasts. Therefore, it is unknown if WNT10B affects the osteoclast directly. *Wnt10b*-knockout mice or *Wnt10b* overexpressing mice (osteocalcin-Cre) have no differences in osteoclast numbers compared to wild-type mice (Bennett et al., 2007; Stevens et al., 2010), although the *Wnt10b* overexpressing mice have an increase in the bone resorption marker CTX (a collagen metabolite produced by type I collagen degradation due to osteoclast activity).

### 4.1 Regulation of WNT10B in bone

RUNX2 is considered the master transcription factor of osteoblasts, as it is necessary for osteoblast differentiation and induces many other osteoblast differentiation genes. WNT10B upregulates *RUNX2* expression (Bennett et al., 2005), and transcription factors downstream of WNT signaling (β-catenin and TCF1) bind directly to the *RUNX2* promoter (Gaur et al., 2005). In addition, *WNT10B* is a direct target of *RUNX2*, as RUNX2 binds to the *WNT10B* promoter, overexpression of RUNX2 increases *WNT10B* expression, and knockout of *RUNX2* in osteoblasts decreases *WNT10B* expression (Qin et al., 2019; Qin et al., 2021). CBFβ, a non-DNA-binding partner of Runt-related transcription factors (RUNX1, RUNX2, and RUNX3), is also recruited to the promoter of *WNT10B* (Wu M. et al., 2017) to upregulate the transcription of *WNT10B*. *Cbfβ*-knockout mice have a low bone-mass phenotype similar to *WNT10B* knockout mice.

GATA4 binds at the promoter of *WNT10B* and at a downstream enhancer, as discovered by ChIP-sequencing. Furthermore, knockout of *Gata4* decreases *Wnt10b* expression, corresponding with a decrease in trabecular bone properties. GATA4 also transcriptionally regulates other components of the WNT signaling pathway, including *Wnt3a*, *Fzd6*, and *Dkk1* (Khalid et al., 2021; Suthon et al., 2022b).

BMP9, an osteogenic bone morphogenic protein (BMP) family member, induces phosphorylation of SMAD1/5/8, which binds to the promoter of *Wnt10b*, along with pCREB, leading to the upregulation of *Wnt10b*. Furthermore, the combination of BMP9 and WNT10B increases the expression of *Runx2* and alkaline phosphatase (ALP) and induces mineralization, compared to either factor alone (Liao et al., 2019; Liang et al., 2020). Conversely, PTEN, which is anti-osteogenic, can decrease BMP9-mediated increases in *Wnt10b* expression (Li et al., 2020a).

Post-transcriptionally, *WNT10B* is regulated by microRNAs (miRNAs) and long non-coding RNAs (lncRNAs, Table 1). The expression of maternally expressed gene 3 (MEG3), a lncRNA, is upregulated in bones with a non-union fracture and binds to the promoter of *Wnt10b* to suppress its expression. Silencing of MEG3 leads to an increase in WNT10B protein levels and faster fracture healing (Liu Y. B. et al., 2019). miR-33b-5p binds to the 3’ end of *WNT10B* mRNA to regulate its protein expression. The expression of long intergenic non-protein coding RNA 2349 (linc02349) increases during osteogenesis and acts as a molecular “sponge” for miR-33b-5p, thus increasing the levels of *WNT10B* (Cao et al., 2020). In comparison, the knockout of *Dicer1*, an endoribonuclease in the miRNA maturation process, led to a decrease in *Wnt10b* expression and less osteoblast differentiation (Wu H. Y. et al., 2019).

**TABLE 1:** *WNT10B* regulation *via* non-coding RNA/miRNA.

RNA type	Name	Tissue/disease	Upstream/downstream mediator	Reference
circRNA	CTB-193M12.5	Hepatocellular carcinoma	CTB-193M12.5 → NSD1 → WNT10B	Zhang et al. (2022)
lncRNA	LINC00926	PBMCs	LINC00926→ WNT10B	Bam et al. (2022)
lncRNA	KB-68A7.1	Hepatocellular carcinoma	KB-68A7.1 ⊣ NSD1 → WNT10B	Zhang et al. (2021b)
lncRNA	Maternally expressed gene 3 (MEG3)	Bone	MEG3 ⊣ WNT10B	Liu et al. (2019b)
miRNA	miR-15b-5p	Skin (dermal papilla cells)	LncRNA-599547 ⊣ miR-15b-5p ⊣ WNT10B	Yin et al. (2022)
miRNA	miR-16	Lung fibrosis	miR-16 ⊣ WNT10B	Kadota et al. (2021)
miRNA	miR-33b-5p	Bone	Linc02349 ⊣ miR-33b-5p ⊣ WNT10B	Cao et al. (2020)
miRNA	miR-148a	Adipocytes, lung fibrosis, pancreatic cancer, OSCC, colon adenocarcinoma, and endometrial carcinoma	miR-148a ⊣ WNT10B	Cho et al. (2013), Cho et al. (2016), Kadota et al. (2021), Li et al., 2021a, Min et al. (2016), Peng et al. (2017), Shi et al. (2019), and Aprelikova et al. (2013)
lncRNA/miRNA	HOTAIRM1/miR-148a	Thyroid cancer	HOTAIRM1 ⊣ miR-148a ⊣ WNT10B	Li et al. (2021a)
miRNA	miR-329	Skin (dermal papilla cells)	PCAT1 ⊣ miR-329 ⊣ WNT10B	Lin et al. (2020)
miRNA	miR-149-3p	Endometrial carcinoma	HOXB-AS1 ⊣ miR-149-3p ⊣ WNT10B	Liu et al. (2020a)
miRNA	miR-370	Cholangiocarcinoma	IL6 ⊣ miR-370 ⊣ WNT10B	An et al. (2012)
miRNA	miR-885-3p	Lung adenocarcinoma	circTUBGPC3 ⊣ miR-885-3p ⊣ WNT10B	Yang Y. et al. (2021)
miRNA	miR-6777-3p	Hepatocellular carcinoma	LINC00355:8 ⊣ miR-6777-3p ⊣ WNT10B	Zhou et al. (2020)
miRNA	miR-7113-5p	PBMCs	miR-7113-5p ⊣ WNT10B	Bam et al. (2022)

In osteoclasts, calcitonin, a peptide hormone secreted from the parafollicular cells of the thyroid gland that inhibits osteoclastic resorption, has been shown to increase *WNT10B* expression (Hsiao et al., 2020). Similarly, calcitriol (vitamin D3) decreases osteoclast differentiation *in vitro* and simultaneously increases the expression of *WNT10B* (Lu C. L. et al., 2018). Several other factors, including TGFβ and the chronic kidney disease drug cinacalcet, a calcimimetic, also increase the expression of *WNT10B* in osteoclasts (Ota et al., 2013; Zheng et al., 2019).

### 4.2 WNT10B as a therapeutic agent in bone

Non-union fractures occur in 5%–10% of fractures (Panteli et al., 2022). Surgical treatments include bone graft or bone graft substitute, internal fixation, and/or external fixation. Each has advantages and disadvantages, and improved solutions are being investigated. WNT10B has been suggested as a growth factor that can improve bone healing in the following studies. A *WNT10B*-expressing lentivirus was shown to be sufficient to heal an atrophic non-union fracture in rat femurs without the addition of a scaffold or cells (Gao et al., 2015). In comparison, human umbilical cord MSCs that overexpress *WNT10B* resulted in better mineralization and accelerated bone defect healing in a calvarial defect model (Liu et al., 2020g). Similarly, human umbilical cord MSCs transfected with *WNT10B* in hydrogel placed into a femoral fracture accelerated healing. While there was a similar amount of a cartilaginous callus at 2 weeks, WNT10B induced more bone formation 4 weeks after the fracture. WNT10B also induced more angiogenesis at the site of the fracture, which is an important step in fracture healing (Hu et al., 2022). Liu et al. (2018) used adenoviral-delivered *WNT10B* in bone marrow-derived mesenchymal stem cells in an ovariectomized rat model, and the expression of *WNT10B* accelerated osseointegration of a femoral implant (Liu et al., 2018). Hsu et al. (2020) used CRISPR activation of *WNT10B* (along with FOXC2) to overexpress *WNT10B* in bone marrow-derived mesenchymal stem cells to efficiently heal critical-sized calvarial defects in rats (Hsu et al., 2020). Based on these studies, WNT10B optimized for delivery, cell type, and scaffold could potentially be used in humans with non-union fractures to increase bone healing.

In addition, WNT10B could potentially be used to treat osteoporosis. Overexpression of *Wnt10b* in osteoblasts *via* the osteocalcin promoter prevented diabetes-induced bone loss and marrow adiposity but did not affect blood glucose levels. The probiotic *Lactobacillus reuteri (L. reuteri)* also prevented diabetes-induced bone loss while increasing *Wnt10b* expression in a total bone analysis (Zhang et al., 2015).

Furthermore, overexpression of *Wnt10b* in osteoblasts protected against glucocorticoid-induced osteoporosis (GIO), the most common cause of secondary osteoporosis. Glucocorticoids decrease trabecular bone, decrease expression of *Wnt10b*, and alter the gut microbiome. Mice exposed to prednisolone had a decrease in BV/TV (bone volume/total volume) and trabecular properties, but the *Wnt10b* transgenic mice did not have these decreases. The suppression of *Wnt10b* in bone after glucocorticoid treatment was restored by *L. reuteri* (Schepper et al., 2020), as in the diabetes model described previously. In another model of secondary osteoporosis, in which osteoporosis is induced by vitamin A, *Lactobacillus plantarum* HFY15 or *Lactobacillus fermentum* ZS40 isolated from yak yogurt restored BV/TV and other trabecular properties in rats and increased *Wnt10b* levels in the bone (Liu et al., 2020d; Liu et al., 2020f).

Tyagi et al. (2018) showed that the probiotic *Lactobacillus rhamnosus* GG also increased bone mass. The microbial metabolite butyrate, which is a short-chain fatty acid, is produced from probiotics, including *Lactobacillus rhamnosus* GG. Butyrate stimulated bone formation *via* T-regulatory cell (Treg) activation that mediates the upregulation of *Wnt10b* from bone marrow CD8^+^ T cells (Tyagi et al., 2018). Mechanistically, the increase in butyrate-activated Tregs promoted the assembly of a NFAT1–SMAD3 transcriptional complex to the *Wnt10b* promoter in CD8^+^ T cells, which drove the activation of the T cells to secrete WNT10B. The authors reduced the number of Tregs using anti-CD25 antibodies or in reconstitution experiments using either the TCRβ^−/−^ or CD8^+^ T cells from *Wnt10bKO* mice, which prevented the butyrate-mediated bone formation effects. Mechanistically, this work demonstrated that the probiotic increased butyrate production in the gut and that the butyrate increased the expression of *Wnt10b* specifically in CD8^+^ T cells compared to the Zhang et al. (2015) and Schepper et al. (2020) studies that analyzed whole bones, which included many cell types. Butyrate was also required for intermittent parathyroid hormone (iPTH) to increase the number of Tregs and Treg production of WNT10B (Li et al., 2020b). Therefore, the actions of iPTH and WNT10B are paracrine effects of T cells on osteoblasts (Tyagi et al., 2018).

## 5 WNT10B and the immune system

WNT signaling has been shown to regulate a vast array of biological functions in the immune system, including hematopoietic stem cells (HSCs), normal and malignant hematopoiesis, self-renewal, proliferation, and terminal differentiation of immune cells (Roozen et al., 2012; Bigas et al., 2020; Roo and Staal, 2020). The transcription factors downstream of WNT signaling, T cell factor 1 (TCF1) (Falk et al., 1983), and lymphoid enhancing factor-1 (LEF1) (Castrop et al., 1995) were first identified in immune cells. Of the 19 WNT family members, 12 play a role in immune cells, notably WNT2B, WNT3A, WNT5A, WNT5B, and WNT10B (Wend et al., 2012).

WNT10B’s main functions in the immune system have been shown to arise from T cells. The thymus is the primary organ for T-cell development. As the cells develop, they undergo a negative and positive selection to ensure appropriate T-cell function and the prevention of autoimmune or autoreactive T cells. This is an intricate and complex procedure by which both thymic stromal cells, most notably dendritic cells, and the thymus epithelia play critical roles in normal T-cell lymphopoiesis. *Wnt10b* mRNA has been detected in embryonic thymic development at day 13 (E13) (Balciunaite et al., 2002). The mouse hematopoietic progenitors have migrated to the bone marrow at this stage, but thymic development has not been completed. Since our last review, important new findings on the role of WNT10B in immune cells are discussed in the following paragraphs.

Parathyroid hormone (PTH) regulates calcium and phosphate homeostasis, which has an impact on bone turnover. Primary hyperparathyroidism (PHP) manifests into an increased risk for bone fractures due to its catabolic effects on bone. In contrast, iPTH therapies using amino acids 1–34 of PTH are used clinically to treat post-menopausal osteoporosis. In 2009, the Pacifici Lab demonstrated that iPTH increases the production of WNT10B in bone marrow CD8^+^ T cells, increasing trabecular bone density (Terauchi et al., 2009), previously reviewed in Wend et al. (2012). A follow-up paper from the Pacifici Lab demonstrated that the ability of iPTH to induce WNT10B expression in T cells is dependent on the parathyroid hormone receptor (PPR). A T-cell-specific PPR disruption (*T-cell PPR^T-cells−/−^* mice) was generated by crossing PPR^flx/flx^ mice with mice expressing *Cre* under the control of a T-cell-specific promoter (*Lck*). Comparisons of PPRflx/flx *versus PPR^T-cells−/−^* with or without iPTH therapies revealed that the *PPR^T-cells−/−^* mice had no increase in bone marrow density in response to iPTH and iPTH failed to induce the production of *Wnt10b*. The results argued that the iPTH influence on T cells to secrete WNT10B protein depends on T cells expressing PPR (Bedi et al., 2012).

Furthermore, iPTH treatment expands hematopoietic stem and progenitor cells (HSPCs), but the underlining mechanism has been unknown. Li et al. (2012) demonstrated that T cells were required for the expansion of short-term HPSC (ST-HPSCs) mediated by iPTH, as this effect was abrogated by the disruption of PPR signaling. Mechanistically, secretion of WNT10B was required from the T cells to expand ST-HPSCs. Concurrently, WNT10B also activated WNT signaling in bone marrow stromal cells (BM-SCs). In the absence of WNT10B expression in T cells, several bone marrow stromal cell genes (*Ahr*, *Axin2*, *Cyr61*, *Nkd2*, *Tagin*, *Tgfβ3*, *Thhbs1*, *Twist1*, and *Wisp1*) were not induced by iPTH therapies. These data prove that WNT10B secretion from the T cell is necessary for ST-HSPC expansion after iPTH therapies (Li et al., 2012).

In a follow-up manuscript, Li et al. (2013) showed that ovariectomized (OVX, mice lacking ovaries and consequently, estrogen production) female mice expanded ST-HPSCs, which was dependent on the T-cell costimulatory molecule CD40 ligand (CD40L). T-cell production of WNT10B required the expression of CD40L for OVX mice to expand ST-HPSCs and activate bone marrow stromal cells. This model would suggest that the use of antiestrogens would expand ST-HPSC function. Moreover, this activity requires CD40L expression on the T cells for the secretion of WNT10B (Li J. Y. et al., 2013). Robinson et al. (2015) demonstrated that T-cell expression of CD40L potentiated the activity of iPTH bone anabolic activity. CD40L^−/−^ mice had decreased secretion of WNT10B from T cells, thus causing iPTH to be unable to increase trabecular bone density. Moreover, bone marrow stromal cells devoid of CD40L failed to upregulate the WNT10B-dependent WNT targets after iPTH therapy. This paper argues that CD40L expression is required for iPTH to induce the secretion of WNT10B from T cells (Robinson et al., 2015).

D’Amelio et al. (2015) evaluated the role of WNT10B production in T cells after iPTH therapy in a randomized trial of 82 women diagnosed with either osteoporosis or PHP. The randomized group included women treated with vitamin D and calcium alone (*n* = 22), 1–84 PTH (*n* = 42), or bisphosphonate ibandronate (*n* = 18). The results from the randomized trial indicated that patients with PHP did not increase *WNT10B* compared to healthy controls. In contrast, iPTH therapy increased *WNT10B* expression in T cells, compared to ibandronate treatment (D'Amelio et al., 2015). This was the first time it was shown that in humans, iPTH treatment increases WNT10B production in T cells but not in B cells or monocytes.

Cyclic adenosine monophosphate (cAMP) is generated following the engagement of the T-cell receptor (TCR) with antigen presented by antigen-presenting cells. cAMP-dependent phosphodiesterase (PDE) inhibitors such as pentoxifylline (PTX) can activate cAMP signaling. PTX, which activates cAMP signaling to downstream effectors PKA, had been recognized to increase bone formation, the mechanism of which was unknown. In a preclinical model, Roser-Page et al. (2022) used PTX to investigate bone turnover to determine if the mechanism of action was through T cells secreting WNT10B. PTX induced WNT10B production in CD3- and CD28-activated T cells. Suppression of cAMP mediator protein kinase A (PKA) decreased *Wnt10b* expression in the T cells. The increase in bone mass mediated by PTX occurs when T cells are activated.

WNT10B has differential expression in male and female immune cells. In wild-type CB57L/6 mice, the percentage of WNT10B-positive myeloid cells was higher in females than males. In contrast, males had higher levels of WNT10B-positive cells in lymphoid cells. Specifically in males, the absence of TNFα increased bone marrow WNT10B expression but significantly reduced the number of WNT10B-positive dendritic cells, CD4^+^ T cells, CD8^+^ T cells, macrophages, and granulocytes. These results suggest a possible role for TNFα activity in regulating the expression of WNT10B-positive immune cells. In contrast, ovariectomy reduced the expression of WNT10B in bone marrow cells, further demonstrating a role for estrogen and sex-specific regulation of WNT10B (Collins et al., 2017).

Periodontal disease (PDD) is a gum disease of the surrounding bone that supports the teeth and is mostly seen in adults. Wolf et al. (2016) used human periodontal ligament (hPDL) cells, which can exhibit osteoblastic properties, to model inflammatory PDD (Wolf et al., 2016). hPDL cells from the third molar, isolated from healthy subjects aged 12–14 years with no signs of periodontitis, and enriched CD8^+^ T cells, isolated from matched individuals, were either cultured alone or in combination and exposed to PTH (1–34). *WNT10B* expression was observed only in the T cells when PTH was added. hPDL cells were treated with recombinant WNT10B (rWNT10B) to measure the direct effects of WNT10B on the hPDL cells. rWNT10B marginally increased the proliferation of the primary hPDL cells, and over 3 days, the hPDL cells had increased osteoblast differentiation markers (alkaline phosphatase and osteocalcin protein levels). A co-culturing system with both the T cells and hPDL plus PTH (1–34) increased alkaline phosphatase and osteocalcin relative to control. A WNT10B neutralizing antibody reversed the effects mediated by PTH.

In addition to affecting bone mineral density, probiotics have been shown to prevent obesity and modulate the immune system. The probiotic bacterium *Clostridium butyricum* (*CB*) produces butyrate, which is known to inhibit pro-inflammatory cytokines. Li H. et al. (2022) evaluated the effects of CB on fat deposition (Li H. et al., 2022). CB administration increased blood butyrate suppressing adipogenic markers and size distribution of inguinal white adipocyte tissue (iWAT) and epididymal WAT (eWAT). Butyrate effectively increased anti-inflammatory peripheral regulatory FoxP3^+^ T cells (Arpaia et al., 2013). Li et al. (2022) showed an increased number of FoxP3^+^CD4^+^ Tregs after CB administration in iWAT tissue. FACS-sorted Tregs had an increase in *Wnt10b* mRNA levels. CB administration had no effects on fat accumulation in *Wnt10bKO* mice. These results determined that *CB’s* ability to block fat accumulation requires the secretion of WNT10B protein from Tregs. The role of WNT10B in adipocytes is further described in Section 8.

Rheumatoid arthritis (RA) is an inflammatory autoimmune disease that causes crippling disabilities in over 1.3–1.5 million people affected by this disease in the United States. RA-associated bone loss, and cartilage destruction is mediated by autoantigen autoreactive T cells (Fournier, 2005). In RA, T cells infiltrate the synovial membranes and initiate macrophages and synovial fibroblasts to transform into a tissue-destructive force. CD3 and CD28 mediate T-cell activation. A CD28-costimulatory pharmacological inhibitor abatacept (CTLA-4Ig), which is FDA-approved for intractable RA, causes T-cell dormancy (T-cell anergy), a form of perpetual tolerance after antigen interaction. Roser-Page et al. (2014) determined that abatacept increases bone mass in young (3 months) and mature (6 months) mice. Abatacept-treated mice had 200-fold higher *Wnt10b* mRNA in total bone marrow cells compared to the Ig control, and *in vitro* isolated T cells had a sixfold increase in *Wnt10b* expression compared to control-treated cells. Isolated T cells, activated by CD3, increased *Wnt10b* mRNA. In contrast, when CD3 and CD28 were co-activated, the increase in the *Wnt10b* mRNA expression was reversed. An antigen-presenting cell (APC) assay was conducted with isolated CD11C dendritic cells cultured with ovalbumin-specific peptide to serve as the APC. The challenged DCs were then co-cultured with CD8^+^ T cells expressing ovalbumin-specific TCR cells. A combination of both cell types increased *Wnt10b* mRNA levels over those of the two cell types alone. When abatacept was added to the APC assay, a 20-fold increase in *Wnt10b* mRNA was observed. These results suggest a novel role of antigen-presenting dendritic cells in increasing the levels of *Wnt10b* expression (Roser-Page et al., 2014). Roser-Page et al. (2018) paradoxically determined that, in the absence of *Wnt10b*, there was a decrease in bone mass rather than no change in response to abatacept (Roser-Page et al., 2018). The authors attribute this to an increase in sclerostin (a WNT inhibitor) in the bone marrow stroma of wild-type mice, but this is not shown in the *Wnt10bKO* mice, so this is not convincing.

Asthma is a chronic inflammatory disease that is very common among children and is often associated with the activation of CD4^+^ T-helper 2 cells (CD4^Th2^). Trischler et al. (2016) used an *in vivo* house dust mite (HDM) asthma model. The HDM-treated mice demonstrate upregulation of *Wnt10b* mRNA in the lung and splenocyte-enriched T cells. The *Wnt10bKO* mice were challenged with HDM resulting in an increased number of eosinophils, EMBP protein expression (a marker of activated eosinophils), and *Il4*, *Il13*, *Arg1*, and *Ccl2* mRNA expression over wild-type mice. This signature is consistent with either Th2 polarization or macrophage activation. Subsequently, splenic T cells were isolated from wild-type and *Wnt10bKO* mice, activated *via* CD3/CD28, and cocultured in the presence or absence of IL4 and IL12. *Wnt10b*KO T cells in the presence of IL4 increased GATA3 and IL4. GATA3 and IL4 expression levels are known to be produced from activated Th2. In contrast, *Wnt10bKO* T cells cultured in the presence of IL12 did not upregulate the Th1-promoting marker T-bet mRNA. Moreover, *Wnt10bKO* HDM-challenged mice increased effector T cells (CD4^+^CD44^hi^CD62L^lo^, CD4^+^CD69^hi^CD11a^hi^, and CD8^+^CD44^hi^CD62L^lo^). The results suggest that WNT10B expression regulates type 2 inflammation and the activation of a Th2 response in HDM-challenged mice.

Post-traumatic stress disorder (PTSD) frequently occurs in the aftermath of a psychologically traumatic event. PTSD is associated with a heightened inflammation that can be assessed by determining the levels of the inflammatory chemokines IL17A and interferon-gamma (IFNγ) in a patient’s peripheral blood mononuclear cells (PBMC). Bam et al. (2020) demonstrated increased levels of WNT10B in PTSD patients compared to healthy controls. miR-7113-5p was identified by microRNA expression analysis to be downregulated in PTSD patients and decreased the levels of WNT10B protein expression in a monocyte cell line (Table 1). rWNT10B treatment in human PBMCs in combination with PMA increased IFNγ expression significantly, relative to PMA treatment alone (Bam et al., 2020). In a follow-up study to determine how WNT10B is upregulated in PTSD, Bam et al. (2022) conducted RNA-sequencing on PBMCs of PTSD patients and determined that LINC008926 was upregulated compared to controls (Table 1). An increase in the open chromatin mark H3K4me3 at the promoter of WNT10B is due to LINC00896 recruiting a histone methyltransferase (MML1) onto the promoter of *WNT10B*. Knockdown experiments of MML1 showed a loss of *WNT10B* transcripts and a subsequent reduction in IFNγ. Conversely, the authors knocked down KDM5B (a histone demethylase) and increased the levels of the *WNT10B* transcript. rWNT10B added to preactivated PBMCs from healthy donors increased IL17A and IFNγ expression. ICG-001, which blocks CBP-mediated acetylation of β-catenin preventing active gene expression, significantly reduced the IL17A and IFNγ expression levels. Knockdown of LINC008926 in PBMCs and the lymphoblastic cell line TALL-107 decreased the *WNT10B* and *IFNγ* expression levels (Bam et al., 2022).

Severe infection elicits an inflammatory response, which causes an estimated 20,000 deaths worldwide, even when antibiotics are used. To better understand humans with sepsis, whole blood was analyzed for the correlation of inflammatory cytokines and WNT ligands. *WNT10B* correlated with the number of monocytes and expression of *IL6* and *TNFα*, but not *IL10*, in patients with sepsis but not in healthy patients. In comparison, mice were administered LPS to induce endotoxemia, a model for human sepsis. In splenocytes, *Wnt10b* expression was significantly associated with *Tnfα*, *Il12b*, and *Il10* after 1.5 h of LPS treatment. They further showed that canonical TLR4 signaling events *via* MYD88 mediated Wnt10b mRNA expression in response to LPS. Inhibition of WNT signaling by either ICG-001 or IWP-2 (a porcupine inhibitor, thereby inhibiting WNT secretion) reversed the cytokine signatures. Therefore, WNT ligands, including WNT10B, are part of the immune response during septic shock (Gatica-Andrades et al., 2017).

## 6 WNT10B and teeth

In 1998, WNT10B was shown to be expressed at the beginning of tooth development in epithelial cells, signaling to the mesenchyme (Dassule and McMahon, 1998). In the past decade, polymorphisms and mutations in WNT10B have been associated with tooth abnormalities.

Tooth agenesis is a developmental absence of certain teeth. Up to 20% of people are missing their third molar. An SNP (rs833843) in the 5’ promoter of *WNT10B* correlates with tooth agenesis (Magruder et al., 2018). The T allele at rs833843 was shown to have less transcriptional activation activity in a luciferase reporter assay compared to the C allele (Williams et al., 2021), suggesting that WNT10B levels are critical for tooth formation in humans.

Oligodontia is a severe form of tooth agenesis in which at least six teeth are missing. Four different coding mutations in *WNT10B* (p.Arg211Gln, p.Pro190Arg, p.Trp262^∗^, and p.Phe284Cys) have been detected in families with oligodontia (Yu et al., 2016). *In vitro* expression of the mutant ligands decreased the activation of the TCF-luciferase reporter gene and decreased endothelial differentiation of dental pulp stem cells. The p.Arg211Gln mutation is predicted to decrease the binding affinity of the WNT10B ligand to FZD8, p.Trp262^∗^ is a truncating mutation, and the other two mutations have unknown consequences (Yu et al., 2016). In a second study, Kantaputra et al. (2018) found that *WNT10B* mutations are associated with oligodontia, microdontia (small teeth), short tooth roots, dental pulp stones, and taurodontism (elongation of the pulp chamber of the tooth) (Kantaputra et al., 2018). Mutations in other components of WNT signaling (WNT10A, LRP6, and KREMEN1) have also been shown to lead to tooth agenesis (Chu et al., 2021).

## 7 WNT10B and split-hand/foot malformation

SHFM is a congenital limb malformation characterized by the absence of certain digits, leading to a claw-like hand or foot. Mutations in *WNT10B* and several other genes have been shown to cause SHFM, with *WNT10B* mutations associated with SHFM type 6. WNT signaling plays a role in many stages of limb development during embryogenesis, and specifically, *WNT10B* is expressed in the limb bud at all stages (Wang and Shackleford, 1996). Mutations in *WNT10B* in patients with SHFM were first identified in a Turkish family in 2008 (c.994C→T, p.R332W) (Ugur and Tolun, 2008). Since then, several other case studies have shown a variety of *WNT10B* mutations in SHFM individuals (Khan et al., 2012; Ullah et al., 2018; Khan et al., 2019; Al Ghamdi et al., 2020; Bilal et al., 2020; Elalaoui et al., 2021). Many of these result in frameshifts or premature stop codons.

Interestingly, the patients with SHFM do not have tooth abnormalities and have different mutations than those with tooth agenesis (Yu et al., 2016). Individuals with tooth agenesis have mutations in amino acids between 190 and 284, and those with SHFM have mutations between 329 and 388, in addition to non-sense and frame-shift mutations. Furthermore, the knockout of *Wnt10b* in mice does not lead to an SHFM phenotype. *Wnt10b* knockout mice are obese and have low bone mineral density, which has not been reported in SHFM patients. Therefore, a mutated *WNT10B* is functionally different from the absence of *WNT10B*, or there are species-specific differences.

## 8 WNT10B and adipocytes

### 8.1 Function of WNT10B in adipocytes

As previously mentioned in Section 4, WNT10B is often used as a marker of increased osteoblastogenesis or decreased adipogenesis, based on *in vitro* and *in vivo* experiments in the previous decade (Wend et al., 2012). Several gain-of-function mouse models showed the effects of WNT10B in adipocytes: overexpression of *Wnt10b* in adipocytes with the *FABP4* (fatty acid-binding protein 4) promoter (expressed in white and brown adipose tissues) inhibited adipogenesis (Longo et al., 2004) and overexpression of *Wnt10b* with the *UCP1* promoter (expressed highly in interscapular tissue) converted brown adipose tissue to white adipose (Kang et al., 2005). Furthermore, the *FABP4*-*Wnt10b* mice are resistant to diet-induced obesity and do not gain significant bodyweight on the ob/ob background (Wright et al., 2007). *In vitro*, WNT10B decreased the adipogenic differentiation of 3T3-L1 preadipocytes while simultaneously increasing osteoblast differentiation, similar to the overexpression of β-catenin. Mechanistically, WNT10B signals through β-catenin to suppress the expression of PPARγ (a master adipogenic transcription factor) and ID2 (which is known to induce PPARγ expression and adipogenesis) (Cawthorn et al., 2012). WNT10A and WNT6 (Cawthorn et al., 2012), along with WNT1 and WNT3A, have similar roles as WNT10B (Liang et al., 2020) in adipocyte and osteoblast differentiation.

Bone marrow adipose tissue (BMAT) is a distinct form of adipose that regulates skeletal homeostasis and energy metabolism (Pachon-Pena and Bredella, 2022). WNT10B is often used as a marker of adipogenesis in bone marrow adipocytes (Georgiou et al., 2015; Lee et al., 2019), as in other adipose depots. Overexpression of *Wnt10b* in osteoblasts with the OCN promoter (OCN-*Wnt10b*) led to a decrease in BMAT. Calorie restriction increases the percent of BMAT, which is blunted in the OCN-*Wnt10b* mice (Cawthorn et al., 2014). WNT10B’s regulation of BMAT requires further investigation, such as determining if WNT10B represses BMAT with the same mechanism as in white adipose.

### 8.2 Regulation of WNT10B in adipose tissue

Research in the past decade on WNT10B in adipocytes has focused on the transcriptional regulation of *WNT10B*. Adipocytes use several mechanisms to repress *WNT10B* during adipogenesis: DNA methylation, WNT signaling inhibitors, and microRNAs.

During adipogenesis, the promoter of *WNT10B* becomes methylated, and the chromatin is in a closed conformation, preventing gene transcription. The methionine adenosyltransferase MAT2A and the lysine N-methyltransferase EZH2 are recruited to the *WNT10B* promoter during adipogenesis, leading to histone 3, lysine 27 (H3K27) methylation, and gene suppression (Zhao et al., 2018). *WNT10B* promoter methylation can be reversed with the inhibitor 5-azacytidine (Fox et al., 2008). In the absence of methylation, CREB regulates *WNT10B* expression. However, in the presence of methylation, CREB is prevented from binding the promoter, and *WNT10B* expression is repressed in adipogenesis. In addition to two CREB response elements (CRE), there are three HIF-responsive elements (HRE) in the promoter of *WNT10B* bound by HIF-2α. Hypoxia increases *WNT10B* expression and subsequently decreases adipogenesis (Park et al., 2013).

Another factor that decreases WNT10B levels in adipogenesis is microRNA-148a (miR-148a, Table 1). The expression of miR-148a increases in response to XBP1, a pro-adipocyte transcription factor, during adipogenesis, corresponding with a decrease in *Wnt10b* expression. miR-148a can bind to the 3′-UTR of *Wnt10b* mRNA and decrease the expression of *Wnt10b* (Cho et al., 2013; Cho et al., 2016).

Dickkopf (DKK) genes DKK1-4 encode secretory proteins that can antagonize WNT/β-catenin signaling by inhibiting WNT coreceptors LRP5 and LRP6 (Niehrs, 2006). The addition of recombinant DKK1 to human adipose-derived stem cells increased adipogenesis *via* upregulation of PPARγ and C/EBPα, with a corresponding decrease in WNT10B mRNA and protein. The addition of the GSK3β inhibitor SB 216763 downregulated DKK-1, activated canonical WNT signaling, and increased *WNT10B* expression (Lu et al., 2016). Thus, the activation of WNT signaling regulates *WNT10B* expression in a feed-forward loop, but this mechanism remains unclear.

*WNT10B* expression in adipocytes can also be regulated differentially by SNPs. In 2011, an SNP (rs833840) in the promoter of *WNT10B* was associated with body fat mass in Korean females (Kim et al., 2011). Subsequently, a genetic association between *WNT10B* polymorphisms and obesity in Belgian males was shown for three *WNT10B* SNPs (rs833841, rs4018511, and rs10875902) (Van Camp et al., 2012). The first (rs833841) is in linkage disequilibrium with the SNP found in the Korean study (rs833840). The other two SNPs are in the 3’ non-coding region of *WNT10B*. The same group investigated two additional populations and did not find the same association with BMI or adiposity. However, they did identify a correlation with bone mineral density (Van Camp et al., 2013). Thus, *WNT10B* polymorphisms may occur in different populations but appear at low frequencies in the published studies, limiting statistical significance, if any.

In summary, as evidenced by transgenic and knockout mice, *in vitro* experiments, and human polymorphisms, WNT10B, *via* canonical WNT signaling, is considered an inhibitor of adipogenesis, and *WNT10B* expression must be inhibited to allow adipogenesis.

## 9 WNT10B and muscle

*WNT10B* is expressed in muscle cells. Myoblasts can differentiate into myocytes or adipocytes, and WNT10B signaling regulates this process. *Wnt10b* knockout mice have increased adipogenesis in actively regenerating myofibers (Vertino et al., 2005). This balance between myocytes and adipocytes was also demonstrated in the rotator cuff. There is increased adipogenesis in muscle tissue near a torn rotator cuff, and this is a prognostic factor for poor recovery after rotator cuff surgery. The expression of *WNT10B* is decreased in the muscle, corresponding with increased adipogenesis, after rotator cuff injury in a rabbit model and human patients (Shah et al., 2017; Kuwahara et al., 2019).

Additional evidence that showed the inverse biology between adipose and muscle was presented in a study comparing patients with high insulin sensitivity (IS) to patients with low IS. Adipose tissue from the high-IS group had a higher expression of *WNT10B* than the low-IS group. In contrast, the skeletal muscle from the high-IS had lower *WNT10B* expression than the low-IS group (Karczewska-Kupczewska et al., 2016).

Intramuscular fat (IMF) is an important characteristic of beef quality and taste, and castration increases the fat content. The muscle of castrated cows had a decrease in WNT10B expression compared to intact bulls. In castrated cows, there was an inverse correlation between *WNT10B* (and β-catenin) expression and fat content (Jeong et al., 2013). Therefore, Jeong et al. (2013) suggested that the WNT10B/β-catenin signaling pathway could be used to predict beef quality. Another study showed that *WNT10B* is higher in the longissimus thoracis muscle (used for ribeye steaks) compared to subcutaneous fat in an age-dependent manner (Soret et al., 2016). *WNT10B* expression did not vary between four different muscles with different IMF levels (Martinez Del Pino et al., 2017). Therefore, further work on the level of *WNT10B* and other genes in muscle and the relationship to IMF should be performed to optimize beef quality.

IMF is also a consideration in donkey meat. Guangling donkeys are raised in China for consumption. RNA-sequencing was performed on high- and low-fat Guangling donkey muscles. One of the most important regulated genes, based on co-expression networks, was *WNT10B*. WNT10B had increased expression in the high-fat group, in contrast to the expected inverse correlation in studies on humans and cows (Li W. et al., 2022) (see Section 8).

Wnt10b in muscle has also been studied in the zebrafish model. Wnt10b signaling inhibits the synthesis of fatty acids in zebrafish myocytes (Liu D. et al., 2019). Knockdown of *Wnt10b* mRNA led to an increase in triglyceride, total cholesterol, and non-esterified fatty acids in the muscle and corresponding increases in the expression of fatty acid synthetase, acetyl-CoA carboxylase, and ATP-citrate lyase. Wnt10b signaling is activated by vitamin E, an antioxidant, and Wnt10b regulates the activities of antioxidant enzymes (superoxide dismutase, peroxidase, and glutathione peroxidase) in the muscle of zebrafish (Liu et al., 2020b).

## 10 WNT10B and fibrosis

Fibrosis is the development of fibrous connective tissue as a reparative response to injury or damage. It is characterized by chronic inflammation, excessive accumulation of mesenchymal proteins [e.g., α-smooth muscle actin (α-SMA), vimentin, fibronectin, and fibroblast-specific protein 1 (FSP-1)], and an increase in the extracellular matrix that disrupts the normal tissue function. WNT signaling plays a role in fibrosis (Hu et al., 2020). In 2011, it was reported that the ectopic expression of *Wnt10b* in skin fibroblasts caused enhanced dermal fibrosis. In addition, in the past decade, further work in systemic sclerosis (SSc) and fibrosis in additional tissues [heart (see Section 12), lungs, liver, and penis] demonstrate a function for WNT10B, specifically in fibrosis.

WNT10B expression correlates with lung fibrosis. The addition of WNT10B increased the expression of mesenchymal markers and epithelial-to-mesenchymal transition. Conversely, the reduction of *WNT10B* by siRNA decreased mesenchymal markers and pulmonary fibrosis (Yang Z. et al., 2021). Furthermore, a reduction in *WNT10B* levels by miR-16 and miR-148a (Table 1), found in extracellular vesicles from the bronchial epithelium, decreased myofibroblast differentiation (Kadota et al., 2021).

WNT pathway genes (including *Wnt10b*) are upregulated in activated hepatic stellate cells leading to liver fibrosis. The mechanism includes repression of PPARγ and liver adipogenesis (Cheng et al., 2008). MSCs reduce liver fibrosis, and the treatment of liver fibrosis in rats with exosomes from MSCs decreased *Wnt10b* expression (Rong et al., 2019). A WNT antagonist also reduced liver fibrosis. In addition to WNT10B, other WNTs, including WNT1, WNT3/3A, WNT4A, and WNT5A, are implicated in liver fibrosis (Cheng et al., 2008).

Fibrosis is also present in penile cavernous tissue from diabetic mice and correlates with erectile dysfunction. This tissue has an increase in *Wnt10b* expression, along with an increase in the expression of four other WNT ligands. TGFβ also increased the expression of *Wnt10b* (Shin et al., 2014). Because the downregulation of *Wnt10b* with small interfering RNA did not decrease the production of extracellular matrix proteins in the fibroblasts, additional research on the role of WNT10B in this system should be performed.

## 11 WNT10B in skin and hair

The skin is the largest organ in the body and consists of appendages that include hair follicles, sebaceous glands, sweat glands, and nails. Many WNT ligands are expressed in the skin and the hair follicle (Wend et al., 2012). Importantly, the earliest and highest expressed WNT ligand in hair follicle development and hair cycle induction is *Wnt10b* (Reddy et al., 2001). Hair follicles have periodic stages: the anagen (growth stage), catagen (regression), and telogen (resting) stages. *Wnt10b* is only expressed in the anagen stage (Li Y. H. et al., 2013; Bai et al., 2021). Specifically, *Wnt10b* is restricted to follicular epithelial cells overlying the dermal condensate (Reddy et al., 2001; Hawkshaw et al., 2020). Overexpression of *Wnt10b* induces the telogen hair follicle to proceed into anagen earlier (Li Y. H. et al., 2013). Adenoviral *Wnt10b* increased the number of proliferating cells and, subsequently, the size of the anagen hair follicle (Lei et al., 2014). Increased proliferating CD34^+^ stem cells were also observed in the bulge region and the outer root sheath of the hair follicle.

Dermal papilla cells are specialized fibroblasts located in hair follicles that work as a signaling center for hair growth. WNT10B promotes the proliferation of cultured dermal papilla cells from mice, Angora rabbits, and Rex rabbits *in vitro* (Ouji et al., 2012; Bai et al., 2019; Wu et al., 2020). Cultured dermal papilla cells with WNT10B had the greatest hair follicle induction in skin reconstitution assays (Ouji et al., 2013). Together these papers demonstrate that WNT10B is produced by the epithelial cells, acting upon the fibroblasts in a paracrine manner. *AXIN2*, the canonical WNT target gene, while expressed in the epithelial cells, is highest in the mesenchymal cells in the early anagen (Hawkshaw et al., 2020).

WNT10B also plays a role in the differentiation of melanocytes (melanin-producing cells) of the hair bulbs during the anagen stage. Intradermal injections of adenoviral *Wnt10b* in a mouse led to more melanocytes with more pigmentation. Similar results were obtained when whisker follicles were treated with adenoviral *Wnt10b*. Adenoviral *Wnt10b* also increased the differentiation of a melanocyte cell line in culture (Ye et al., 2013).

WNT10B is potentially involved in the development of apocrine sweat glands. Apocrine sweat glands are found in hairy regions such as the axilla and secrete nutrients to the opening of hair follicles. While mice and chickens do not have these, sheepskin sweat glands show some similarities to human glands. Examining the complex network of development and maturation of apocrine sweat glands has shown that *WNT10B* has decreased expression in the gland budding stage, compared to the pre-gland stage, as determined by RNA-sequencing. In contrast, *WNT16*, *WNT5A*, and *β-catenin* are upregulated in the gland budding stage (Li S. et al., 2018). Functional studies were not performed.

The role of WNT10B in the skin is further elucidated by examining skin pathologies, such as SSc and psoriasis. SSc is a chronic connective tissue disorder causing fibrosis of the skin and other organs due to overactive fibroblasts. Fibroblasts of those with SSc contain higher levels of nuclear β-catenin, which correlates with increased expression of *Wnt1* and *Wnt10b* (Beyer et al., 2012). Transgenic mice in which *Wnt10b* is expressed from the fatty acid binding protein 4 (*FABP4*) promoter have increased skin thickness and are used to study SSc and anti-fibrotic therapies (Wei et al., 2011). For example, inhibition of the X-linked inhibitor of apoptosis protein (XIAP) reduced the skin thickness of *Wnt10b* transgenic mice (Bergmann and Distler, 2016).

Psoriasis is a chronic inflammatory skin condition causing patches of itchy, flaky skin with scales (lesions). Samples from the lesional skin of psoriasis patients show significantly decreased *WNT10B* gene expression compared to the non-lesional skin of patients and skin from healthy individuals. *WNT7B* showed a similar gene expression pattern, and TCF7L2, a transcription factor downstream of WNT signaling, decreased in the lesional skin compared to non-lesional skin. The gene expression of *WNT10B* and *WNT7B* increases after treatment with narrowband UV, which is a therapy for psoriasis. IL-17 acts on keratinocytes and releases inflammatory mediators that lead to psoriasis symptoms. IL-17A inhibits WNT signaling in bone (osteopenia and osteoporosis have been associated with psoriasis). Although this has to be shown in the skin yet, psoriasis patients have increased IL-17, which could explain the low WNT expression in lesional skin. Current psoriasis therapy incudes IL-17 inhibitors, which not only reduce IL-17 signaling but also may help increase downstream WNT signaling to reduce inflammation (Assarsson et al., 2019).

Agents to increase hair growth could be used to treat various conditions, such as alopecia. Increasing the expression of *WNT10B* either directly or indirectly could induce hair growth. Several hormones and growth factors have been shown to regulate *WNT10B*, including androgens, melatonin, hepatocyte growth factor (HGF), vitamin D, and bone morphogenic protein 6 (BMP6).

Androgenetic alopecia is the most common type of hair loss in men. The androgen DHT decreases *WNT10B* expression in dermal papilla cells while increasing *DKK1* expression. DHT decreases hair follicle stem cell differentiation *in vitro*, and the addition of rWNT10B reverses this effect (Leiros et al., 2017).

HGF from dermal white adipose tissue induces *WNT10B* expression in dermal papilla cells (Nicu et al., 2021). The topical application of small-molecule antagonists to c-Met (the receptor for HGF) for excessive hair growth has been suggested. This is clinically possible as c-Met antagonists have been approved for cancer.

Aoi et al. (2012) showed that vitamin D3 increased *WNT10B* expression in dermal papilla cells; however, vitamin D decreased their proliferation, and WNT10B has been previously shown to increase their proliferation. These conflicting results need to be clarified. All-trans retinoic acid upregulated *WNT10B* expression in a screen of 20 growth factors (Aoi et al., 2012).

Furthermore, BMP6 regulates the hair cycle and is expressed at the highest level in early anagen. BMP6 was shown to repress *WNT10B* expression and decrease the proliferation of hair follicle stem cells, and conversely, WNT10B repressed BMP6 expression and increased the number of proliferating hair follicle stem cells (Wu P. et al., 2019).

The promoter of *WNT10B* is DNA methylated in the catagen and telogen stages, whereas there is a decrease in methylation at the anagen stage, correlating with *WNT10B* expression. Furthermore, there is an increase in histone H3 acetylation, a marker of open chromatin, only during the anagen stage (Bai et al., 2021). The transcription factors that regulate the expression of *WNT10B* at the *WNT10B* promoter in hair follicles remain unknown.

In dermal papilla stem cells, *WNT10B* mRNA expression is downregulated by miR-329 (Table 1). The long non-coding RNA *PCAT1* sponges miR-329 to allow the expression of *WNT10B*. The upregulation of *PCAT1* or a decrease in miR-329 corresponded with enhanced *WNT10B* expression and increased the proliferation of the dermal papilla stem cells (Nicu et al., 2021).

*WNT10B* is also regulated by miR-15b-5p in dermal papilla cells, which in turn is sponged by lncRNA-599547 (Table 1). This long non-coding RNA was shown to be expressed in the secondary hair follicles of cashmere goats at anagen, thus allowing for the expression of *WNT10B* at anagen (Leiros et al., 2017).

*WNT10B* expression can be increased by melatonin, a hormone induced in the brain in response to light and dark. Seasonally, there are changes in the length of the day and, subsequently, the amount of melatonin released. Interestingly, the administration of melatonin can increase *WNT10B* mRNA and protein levels and improve the cashmere yields from cashmere goats, which produce it seasonally (Liu et al., 2021). While melatonin increased the expression of *WNT10B*, it remains unclear if this is a direct or indirect effect.

## 12 WNT10B and the heart

WNT10B expression in the heart was detected along with the cloning of the human gene in 1997. In fact, the tissues with the highest expression levels of WNT10B included the heart (Hardiman et al., 1997). Only in the past decade has there been functional data about the role of WNT10B in the heart, along with an association with fibrosis and disease.

WNT10B is expressed in the intercalated discs of normal cardiomyocytes, and in ischemic cardiomyopathy patients, WNT10B accumulates along the lateral borders of cardiomyocytes. Furthermore, WNT10B protein is increased in cardiomyocytes after experimental myocardial infarction in mice. Overexpression of *Wnt10b* in cardiomyocytes led to improved cardiac function. After injury, *Wnt10b* overexpression led to increased neovascularization and endothelial cell recruitment. WNT10B in the cardiomyocytes induced canonical WNT signaling (β-catenin accumulation in the nucleus and *AXIN2* expression) in the endothelial cells but not in the cardiomyocytes, demonstrating paracrine signaling. The authors show that *Wnt10b* overexpression led to decreased fibrosis (decreased scar formation and decreased collagen deposition after cardiac repair) (Paik et al., 2015). This contrasts with other studies (described here) on fibrosis in the heart and the other tissues presented in Section 10.

Kamimura et al. (2016) demonstrated that *WNT10B* expression levels in the left ventricle increased in heart failure, which was correlated with fibrosis. Pioglitazone, a PPARγ agonist used for type 2 diabetes mellitus, has cardiac antifibrotic effects in animals and human patients. Pioglitazone inhibited WNT signaling and prevented the development of left ventricle fibrosis (Kamimura et al., 2016). WNT10B has been shown to suppress PPARγ in adipose tissue (see Section 8).

Fibrosis is also observed in autoimmune myocarditis. In a mouse model of autoimmune myocarditis, the angiotensin II/angiotensin receptor (Agtr1a) pathway is activated. *Wnt10b* and *Wnt1* are lower in Agtr1a knockout cells with lower fibrosis. The authors state that reduced WNT signaling explains the reduced profibrotic response of *Agtr1a^−/−^* cells (Czepiel et al., 2022).

Adipose tissue is also found in the heart, and its accumulation contributes to atrial fibrillation. Multipotent mesenchymal epicardium-derived cells, which can differentiate into adipocytes, express WNT10B. WNT10B expression is associated with repressed adipogenesis in these cells, as is seen in other MSCs (Suffee et al., 2017).

## 13 WNT10B and the nervous system

Only one study has shown a role for WNT10B in the nervous system (Tassew et al., 2017). In that work, WNT10B was shown to promote axonal regeneration. WNTs have previously been shown to be in exosomes. Tassew et al. (2017) showed that while fibroblast-derived exosomes are needed for axonal regeneration, WNT10B is not in the exosomes. Instead, WNT10B requires the exosomes for activity. What was present in the exosomes and affected WNT10B activity was not described. However, after the addition of exosomes, WNT10B re-localized to lipid rafts for endocytosis. Exosome-WNT10B induction of neurite outgrowth did not affect β-catenin. Instead, it led to mTOR activation and phosphorylation of S6 ribosomal protein (pS6K), indicating WNT signaling through the non-canonical pathway in neurons (Tassew et al., 2017).

## 14 WNT10B and endothelial cells/angiogenesis

WNT signaling is involved in angiogenesis (Olsen et al., 2017). In particular, a role for WNT10B in angiogenesis and endothelial cells has become apparent. Vascular endothelial growth factor (VEGF) is a key angiogenic growth factor. WNT10B regulates *VEGFA* in umbilical cord mesenchymal stem cells (Liu et al., 2020g) and vascular endothelial growth factor receptor 2 (*VEGFR2*) in endothelial cells ((Paik et al., 2015). Furthermore, the expression of *WNT10B* and *VEGFA* genes correlate in the subcutaneous white adipose tissue of 80 individuals, and hypoxia increases *WNT10B* and *VEGFA* in adipocytes (Pourdashti et al., 2022).

The vasculature plays a critical role in bone physiology and fracture healing (Owen-Woods and Kusumbe, 2022). Human umbilical cord mesenchymal stem cells transfected with *WNT10B* accelerated bone healing and induced endothelial cells to migrate and form capillary-like structures in a tube formation assay (Hu et al., 2022).

Treatment of corneal endothelial cells with IL-1β resulted in the activation of WNT10B expression and cellular proliferation. NFκB and c-Jun bound to the proximal promoter of *WNT10B* after IL-1β induction. Then, WNT10B stimulated proliferation by upregulating cyclin D1 expression. Co-treatment with a secreted frizzled-related peptide (sFRP), the β-catenin antagonist XAV939, or a disheveled-PDZ domain inhibitor blocked WNT10B signaling and proliferation. The modulation of WNT10B could treat vision loss secondary to corneal endothelial dysfunction (Lee and Heur, 2015).

## 15 WNT10B and the ovary

The biology of WNT10B in the ovary beyond gene expression has not been determined. Single-cell RNA-sequencing was performed on normal mouse ovaries and ovaries from mice exposed to cigarette smoke, as cigarette smoke affects fertility. Nine different cell types were identified, including oocytes. *WNT10B* was specifically expressed only in the granulosa cells, and WNT signaling pathway genes were enriched. Cigarette smoke increased the expression of *WNT10B* mRNA (Li F. et al., 2022). Others have identified *WNT10B* expression in the full-grown oocyte, ovulated oocyte, zygote, and two-cell stage embryo (Harwood et al., 2008). *WNT10B* expression was downregulated in a mouse model of premature ovulatory failure (Ruohonen et al., 2022). Further studies should be carried out to assess the functional implications of *WNT10B* expression in the ovaries.

## 16 WNT10B and placental development

Dysfunctional placental development is associated with several pregnancy complications, including fetal growth restriction, which impacts 3%–7% of pregnancies in the United States. Several key steps are required for the formation of a functional placenta, including implantation in the maternal endometrium, formation of a syncytium for the exchange of food and waste, generation of villi, and remodeling of the maternal spiral arteries. These steps are accomplished by different subtypes of mature, differentiated trophoblasts, such as syncytiotrophoblasts (STBs), proliferating villous cytotrophoblasts (vCTBs), and invasive extravillous cytotrophoblasts (EVTs). Early work from Sonderegger et al. (2007), using differential gene expression analyses of WNT expression, showed that *WNT10B* expression is the highest in first-trimester trophoblasts but not detected in third-trimester samples. Interestingly, examination of human trophoblast cell lines showed the highest *WNT10B* expression in the EVT-like SGHPL-5 cells and in JEG-3 cells derived from choriocarcinoma (Sonderegger et al., 2007). These findings are supported by more recent studies from Takahashi et al. (2021), who found high *WNT10B* expression in EVT-like cell lines and primary EVT (Takahashi et al., 2021).

Recent work has identified functional roles for WNT10B in the CTB, STB, and EVT. Treatment of EVT cell lines with WNT10B induced *CD44* and *MMP9* expression and invasiveness, whereas knockdown of *WNT10B* decreased *CD44* expression and invasiveness. In primary EVT, WNT10B treatment also increased invasiveness (Takahashi et al., 2021). This work is consistent with Wang et al. (2019), showing that the transcription factor glial cells missing 1 (GCM1) binds the *WNT10B* promoter and induces *WNT10B* expression. Secreted WNT10B binds FZD7, inducing EVT migration (Wang et al., 2019). These data suggest an important role for WNT10B in promoting the invasiveness of EVT. Takahashi et al. (2021) claimed activation of the canonical pathway by WNT10B. In contrast, Wang et al. (2019) claimed non-canonical signaling induced by WNT10B, showing no effect of WNT10B on the expression of *AXIN2* and the failure of WNT10B to affect the expression from a β-catenin/TCF luciferase reporter (Wang et al., 2019). Furthermore, they showed the activation of RAC1 by WNT10B stimulation of trophoblast cell lines. In support of these studies, β-catenin transcript expression is reduced in first-trimester EVT compared to CTB (DaSilva-Arnold et al., 2015). Canonical *versus* non-canonical pathway induction by WNT10B in the regulation of EVT remains an open question.

In addition to playing an important role in the invasiveness of EVT, WNT10B regulates the cell fusion of STBs to form a syncytium. STBs are formed through the process of cell fusion of cytotrophoblasts. Forskolin treatment of BeWo trophoblast cells increased cAMP production, PKA phosphorylation, and CREB phosphorylation, which induced cell fusion to form the syncytium. Forskolin also selectively induced *WNT10B* expression, as other WNTs were unaffected (Malhotra et al., 2015). Forskolin induced *WNT10B* expression in JEG3 cells, a different trophoblast cell line (Wang et al., 2019). Interestingly, forskolin induced total β-catenin expression, suggesting a canonical role for WNT10B in cell fusion (Malhotra et al., 2017).

The hormone human chorionic gonadotrophin (hCG) is critical for the maintenance of pregnancy. hCG binds to the G protein-coupled receptor LHCGR to induce cAMP production. Treatment of BeWo trophoblasts with hCG induced the expression of *WNT10B* and cell fusion. Knockdown of *WNT10B* in BeWo trophoblasts reduced cell fusion, formation of active β-catenin, and the expression of syncytin-1, a key cell fusion protein (Malhotra et al., 2017). These data suggest that hCG induction of WNT10B promotes the fusion of the epithelial cytotrophoblast cells to form the syncytium for the exchange of food and oxygen. Therefore, WNT10B plays key roles in human differentiated trophoblasts that form the functional placenta. Interestingly, the role for WNT10B in murine placental development and function has not been examined. Based on studies in the human placenta, we predict that *Wnt10bKO* mice may be an important tool for understanding the role of WNT10B in the placenta.

## 17 WNT10B and cancer

### 17.1 WNT10B and breast cancer

*WNT10B* was originally discovered as an oncogene in the mammary gland (Lee et al., 1995). Our prior review summarized the biology of WNT10B in mammary stem cells and mouse models of breast cancer (Wend et al., 2012). The last decade of research in breast cancer has produced solid evidence for the oncogenic effects of *WNT10B* in human triple-negative breast cancer (TNBC). WNT10B protein expression was more highly expressed in TNBC (80%) compared to other subtypes of breast cancer (10% in ER+/Her2+/PR+) and significantly predicted worse survival outcomes, tumor size, and grade, suggesting, for the first time, that WNT10B expression is clinically relevant in breast cancer. In contrast, WNT1 expression did not predict survival (Wend et al., 2013). WNT10B overexpression in MDA-MB-231 breast cancer stem cells increased tumor size *in vivo*, and *shWNT10B* decreased the number of mammospheres and colony-forming units compared to controls, further confirming a role for WNT10B in cancer stem cells (Li X. et al., 2022).

*MMTV-Wnt10b* mice generate adenocarcinomas and are a good model for human TNBC (Lane and Leder, 1997; Miranda-Carboni et al., 2008). The most highly upregulated mRNA (by gene expression microarrays) in the *MMTV-Wnt10b* mice, compared to wild-type mice, is high mobility group A family member 2 (*Hmga2*). In contrast, *MMTV-ErbB2* (HER2^+^) tumors do not express HMGA2. During mouse development, the mammary placode is identifiable at embryonic day E11.5, and by E14.5 (early mammogenesis), HMGA2 protein expression was detected in the mammary anlagen bud, epidermis, and mesenchymal tissue. In contrast, *Wnt10bKO* mice did not express HMGA2. WNT10B signaling increases the occupancy of β-catenin at the *HMGA2* promoter, as detected by ChIP analysis, and treating the cells with the WNT inhibitor ICG-001, which blocks CBP-mediated acetylation on β-catenin at Lysine 49 (K49), coincides with the loss of *HMGA2* expression in both mouse and human cells. Therefore, *HMGA2* is a direct target of WNT10B/β-catenin signaling. HMGA2 expression also predicts recurrence-free survival, proliferation, tumor size, nuclear grade, and metastasis in TNBC patients (Wend et al., 2013).

In a follow-up manuscript, El Ayachi et al. (2019) further defined the WNT10B network, composed of β-catenin, HMGA2, and EZH2 signaling. Together, the network predicted a reduction in relapse-free survival and metastasis in chemoresistant TNBC. The expression of HMGA2 and EZH2 was concurrent in *MMTV-Wnt10b*-driven tumors during metastasis. To link this genetically, *HMGA2 KO* mice were backcrossed to the *MMTV-Wnt10b* tumor line and showed loss of EZH2 expression. Mechanistically, HMGA2-EZH2 protein–protein interactions were necessary to maintain lysine 49 (K49) acetylation (K49Ac, activation) of β-catenin and for the displacement of Groucho/TLE1 with TCF-4. TLE1 represses WNT-direct gene target expression by recruiting HDAC to WNT-directed gene expression. CRISPR knockout of both *HMGA2* and *EZH2* in the TNBC cell line MDA-MB-231 blocked primary tumor growth and lung metastasis and restored E-Cadherin *in vivo*. The loss of HMGA2 and EZH2 was necessary to restore a normal mammary epithelial phenotype. Moreover, the protein–protein interactions between HMGA2-EZH2 and EZH2 interaction with β-catenin, TCF4, and LEF1 were preserved in a patient-derived xenograft (PDX) TNBC sample, similar to what was observed in *MMTV-Wnt10b* tumors and the MDA-MB-231 model. HMGA2 was only shown to physically interact with EZH2 but not with WNT-nucleosome proteins (β-catenin, TCF4, and LEF1) (El Ayachi et al., 2019).

The WNT/β-catenin inhibitor ICG-001, or a derivative thereof such as PRI-724, could be used to treat WNT10B-driven tumors. ICG-001 prevented tumor growth and lung metastasis in a WNT10B-expressing TNBC PDX and MDA-MB-231 orthotopic models (El Ayachi et al., 2019; Fatima et al., 2019). Results from *in vitro* work in both mesenchymal-like (ML) TNBC cell lines (MDA-MB-231 and MDA-MB-157) and basal-subtypes (HCC-38 and MDA-MD-468) had similar IC_50_s (half maximal inhibitory concentration) when exposed to the WNT inhibitor ICG-001. The WNT10B direct target genes *HMGA2*, *C-MYC*, *AXIN2*, and *CCND1* were downregulated after ICG-001 therapy. The authors also compared responses to ICG-001 in TNBC PDX cell line models from a naïve chemoresistant PDX (no neoadjuvant therapies) to that of the highly doxorubicin/taxol-chemoresistant PDX. The naïve cell lines were the most sensitive to ICG-001 exposure. In contrast, highly chemoresistant cHCI-10 was the most resistant of all the TNBC cell lines tested. ICG-001 was effective in combination with the conventional cytotoxic chemotherapeutics, cisplatin, and doxorubicin in decreasing the proliferation of MDA-MB-231 cells. WNT10B inhibition combined with FDA-approved therapies could have broad clinical importance.

WNT signaling is a hallmark of TNBC and is associated with specific metastatic pathways (Dey et al., 2013). TNBC can have simultaneous visceral metastases and be associated with poor prognosis if accompanied by bone metastasis (Luo et al., 2017). Fatima et al. (2019) interrogated the WNT10B, β-catenin, HMGA2, and EZH2 signaling axis in highly chemoresistant tumors that metastasize simultaneously to multiple organs, such as the liver, bone, and brain. ICG-001 blocked simultaneous visceral metastasis to bone, liver, ovary, kidneys, and brain in the MDA-MB-231 and HCI-10 TNBC PDX models. Mechanistically, the loss of metastasis was linked to the loss of SNAIL and VIMENTIN protein expression. Furthermore, the combination of ICG-001 and doxorubicin blocked simultaneous multi-organ and bone metastases in the chemoresistant PDX model. These results demonstrate that the addition of a WNT inhibitor to anthracycline therapy can inhibit simultaneous multi-organ metastases (Fatima et al., 2019).

Natural products have provided a direct source of therapeutic agents and have served as the basis for drug development for the past 60 years (Newman and Cragg, 2007). A natural products library of 2,300 compounds was screened for their anti-proliferation and anti-WNT signaling effects on MDA-MB-231 cells (amongst others), and several interesting candidates were identified (Ling et al., 2015; Fatima et al., 2017). The diterpene Jatrophone, derived from the plant *Jatropha isabelli*, interfered with the oncogenic WNT10B/β-catenin/HMGA2 signaling axis and inhibited proliferation in TNBC MSL-subtypes (MDA-MB-231 and MDA-MB-157), BSL-1 subtypes (HCC-38 and MDA-MB-468), and TNBC PDX-derived cells. Mechanistically, JATROPHONE blocked the expression of a WNT reporter (8XTOPFLASH) at the level between receptor complex (LRP6) and β-catenin activation. Jatrophone repressed WNT-direct targets *AXIN2*, *HMGA2*, *C-MYC*, *PCNA*, and *CCND1*. Interestingly, elevated WNT10B expression also coincided with increased resistance to jatrophone exposure in several metastatic cell lines (Fatima et al., 2017). The findings suggested that Jatrophone is poised to further develop as a lead molecule to combat highly chemoresistant metastatic TNBC.

Chen et al. (2022) showed that high expression of the histone lysine methyltransferase NSD1 (nuclear receptor binding SET domain-containing protein 1) could predict the overall survival of breast cancer patients, although the subtype of breast cancer that was analyzed was not discussed. Then, they investigated the role of NSD1 in paclitaxel-mediated drug resistance using parental MCF-7 (ER + breast cancer) *versus* MCF-7 PR (paclitaxel-resistant) cell lines. The MCF-7PR subline had significantly increased NSD1 protein levels. Overexpression of NSD1 in the parental MCF-7 cell line moderately increased proliferation and inhibited apoptosis. The converse was observed when an *siNSD1* was used in the MCF-7PR subline. The authors identified a positive correlation between WNT10B and NSD1 in breast cancer tissue. *siNSD1* decreased the expression of WNT10B protein with concurrent upregulation of H3K27me3 (a mark of transcriptionally inactive chromatin) at the *WNT10B* promoter. The MCF-7 parental line transduced with *siNSD1* had decreased tumor growth *in vivo* relative to the control. Protein analysis of the tumors demonstrated loss of *NSD1*, *WNT10B*, *β-catenin*, *CCND1*, and *C-MYC*. Further studies will be required in the future to validate these findings in models of TNBC (Chen et al., 2022). WNT10B is also regulated by NSD1 in hepatocellular carcinoma (see Section 17.14).

Li et al. (2022) interrogated the effects of sinomenine hydrochloride (SH), which is extracted from a Chinese medicinal plant *Sinomenium acutum*, on breast cancer stem cells (BCSC) (Li X. et al., 2022). SH is known to have anti-inflammatory properties. Exposure of both MCF-7 (ER + breast cancer) and MDA-MB-231 (TNBC mesenchymal stem-like) cell lines to SH led to decreased ratios of CD44^+^/CD24^-^ BCSC markers. SH exposure also decreased the self-renewal capacity of mammospheres, along with migration and invasion in both cell lines. SH decreased the expression of stemness-associated genes (*HMGA2*, *MYC*, and *MET*, amongst others). Other groups have shown that WNT signaling regulates both *MET* and *MYC* expression, and we have shown that both *MYC* and *HMGA2* expression are direct downstream gene targets of WNT10B/β-catenin signaling (Miranda-Carboni et al., 2008; Wend et al., 2013). There is a loss of β-catenin after SH exposure, with a concurrent loss of *WNT10B* mRNA and protein in both MDA-MB-231 and MCF-7 cells. *WNT10B* overexpression reversed the inhibitory effects of SH in mammospheres and colony-forming assays in MCF-7 and MDA-MB-231. SH therapies *in vivo* decreased the tumor growth of BCSCs from MDA-MB-231 cells. The loss of tumor growth coincided with the loss of *WNT10B*, β-catenin, and canonical WNT target genes (*MYC*, *JUN*, *CD44*, and *MET*) in BCSCs. Therefore, SH inhibits TNBC *in vivo* by disrupting WNT10B signaling in BCSCs (Li X. et al., 2022).

Cancer-associated fibroblasts (CAFs) within the stroma of the tumor microenvironment provide epithelial cells with critical regulation of proliferation, invasion, and metastasis and can elicit an anti-immune tumor response. p85α acts as a tumor suppressor, but its functional role in the tumor microenvironment in breast cancer was limited until Chen et al. (2017) described low protein expression of p85α (PIK3R1) in the stroma of stage II and stage III breast cancer. The conditioned media from mouse embryonic fibroblasts with a deletion of p85α increased the proliferation of both 4T1 and MDA-MB-231 breast cancer cells. The fibroblasts also increased the size of tumors and liver metastasis of the breast cancer cell lines. Subsequent analysis showed an increase in EMT markers and a decrease in phosphorylated β-catenin at Ser33/Ser37/Thr41 in 4T1 and MDA-MB-231 cells cultured with the conditioned media from the knockout fibroblasts. The absence of the phosphorylation at Ser33/Ser37/Thr41 indicates engagement of a WNT-canonical ligand (e.g., WNT1 and/or WNT10B)-mediated event. The downstream loss of phosphorylation on β-catenin activates nuclear translocation to activate WNT direct gene targets. WNT10B ligand expression was the highest WNT expressed, >90-fold relative to others, in the absence of p85α in fibroblasts. Supporting the notion that WNT10B was responsible for the stabilization and activation of β-catenin and the increased aggressiveness of both 4T1 and MDA-MB-231 cells, silencing of *WNT10B* by short hairpin RNA in p85α^−/−^ CAFs reversed invasion and tumor growth. Furthermore, an inverse correlation of *WNT10B* mRNA expression and p85α/PIK3R1 was shown in the breast stroma. WNT10B was shown in the exosomes from the p85α knockout fibroblasts. In conclusion, *WNT10B* expression from CAFs in a paracrine manner elicits breast cancer progression (Chen et al., 2017).

### 17.2 WNT10B and pancreatic cancer

At the time of our previous review (Wend et al., 2012), the only mention of WNT10B in pancreatic cancer was at the expression level. Since then, Peng et al. (2017) showed a mechanism of miR-148a in pancreatic cancer progression, signaling through WNT10B. They found that miR-148a (Table 1) is downregulated in pancreatic cancer and that this downregulation correlates with worse survival outcomes and greater rates of lymphatic metastasis (Peng et al., 2017). Furthermore, they reveal that *WNT10B* negatively correlates with miR-148a in these patients. They also show that miR-148a is predicted to bind to the *WNT10B* 3’-UTR, and a miR-148a mimic inhibits *WNT10B* expression. Furthermore, transfecting cells with either a miR-148a mimic or si-*WNT10B* is sufficient to reduce the invasion and migration of a pancreatic adenocarcinoma cell line (BxPC3) (Peng et al., 2017).

### 17.3 WNT10B and thyroid cancer

miR-148a inhibits *WNT10B* not only in pancreatic cancer (Peng et al., 2017), but also in thyroid cancer (Table 1) (Li C. et al., 2021). HOTAIRM1 (HOXA Transcript Antisense RNA Myeloid-Specific 1) is upregulated in patients with thyroid cancer and positively correlates with patient TNM stage and lymph node metastasis. shRNA (small hairpin RNAs) knockdown of HOTAIRM1 markedly increased expression of miR-148a and decreased expression of *WNT10B* in thyroid cancer cell lines (TPC-1 and BCPAP). HOTAIRM1 knockdown significantly reduced cell proliferation and WNT10B protein expression in TPC-1 and BCPAP cell lines, and treatment with an inhibitor of miR-148a rescued these phenotypes. Using the TargetScan database and luciferase assays, they demonstrated that miR-148a directly binds to and suppresses *WNT10B* at the 3′-UTR in thyroid cancer cells. Overall, HOTAIRM1 inhibits miR-148a, which allows for aberrant expression of *WNT10B* in thyroid cancer (Li C. et al., 2021).

### 17.4 WNT10B and oral squamous cell carcinoma

miR-148a also inhibits *WNT10B* in oral squamous cell carcinoma (OSCC) (Table 1). Cancer-associated fibroblasts (CAFs) isolated from OSCC tumors have significantly decreased expression of miR-148a and significant overexpression of *WNT10B* by qPCR compared to normal fibroblasts. Furthermore, WNT10B protein expression is decreased in CAFs transfected with stable miR-148a. Overexpression of miR-148a in CAFs inhibited migration and invasion in an OSCC cell line (SCC-25) by inhibiting WNT10B signaling (Min et al., 2016).

Dai et al. (2019) found that the Homeobox protein Hox-C10 (HOXC10) regulates the expression of WNT10B in OSCC. HOXC10 is overexpressed in OSCC patients by immunohistochemistry and RT-qPCR and correlates with worse overall survival in patients. Furthermore, *HOXC10* knockdown reduced cell migration and invasion in OSCC cell lines (FaDu and SCC4). By using RNA-sequencing, WNT signaling pathway components were identified as targets of HOXC10 signaling. Then, by qPCR, shRNA knockdown of *HOXC10* significantly reduced the expression of *WNT10B* and its downstream signaling components *DVL2* and *LRP5/6* in both FaDu and SCC4 cell lines*.* By immunoblot, they revealed that upon *HOXC10* knockdown, the expression of WNT10B, N-cadherin, and vimentin was downregulated, whereas the E-cadherin expression increased. *In vivo*, loss of HOXC10 decreased tumor size in cell line xenograft models. Furthermore, by immunohistochemistry, *HOXC10*-knockdown tumors had less WNT10B, DVL2, N-cadherin, and Vimentin expression and greater expression of E-cadherin. This study proposes a mechanism by which HOXC10 promotes WNT10B signaling to enhance proliferation, migration, invasion, and epithelial-to-mesenchymal transition in OSCC (Dai et al., 2019).

### 17.5 WNT10B and lung cancer

Furthermore, Yang et al. (2021) reported that *WNT10B* was regulated by non-coding RNAs. Their study identified an overexpression of the circular RNA circTUBGPC3 in lung adenocarcinoma (LAC) patients (Table 1), and high expression of circTUBGPC3 correlated with worse overall survival. As demonstrated by RNA immunoprecipitation, RT-qPCR, and FISH, circTUBGPC3 binds to and inhibits miR-885-3p in SPC-A1 and A549 LAC cell lines. Using the TargetScan database, *WNT10B* was identified as a binding target of miR-885-3p at the 3’-UTR region. The direct binding of miR-885-3p to *WNT10B* was confirmed using luciferase assays with inhibitors and mimics of miR-885-3p. *WNT10B* was also overexpressed in paired LAC and normal tissue and correlated with worse overall survival, lymph node metastasis, and gender. Furthermore, the knockdown of *WNT10B* reduced colony formation, whereas overexpression restored colony formation. Knocking down circTUBGPC3 inhibited *WNT10B* expression while inhibiting miR-885-3p upregulated *WNT10B* in NCI-H460 cells. Overall, this study concluded that circTUBGPC3 promotes lung cancer progression by inhibiting miR-885-3p, thus allowing for the overexpression of *WNT10B* (Yang Y. et al., 2021). Only one paper has investigated WNT10B in the progression of lung cancer, and further work should be carried out to elucidate the actions of WNT10B in normal lung cells, lung fibrosis, and lung cancer.

### 17.6 WNT10B and osteosarcoma

Not only does WNT10B regulate normal bone, but *WNT10B* expression correlates with worse overall survival in osteosarcoma patients (Chen et al., 2008). Research to determine the mechanism by which WNT10B could be affecting osteosarcoma is limited. However, WNT10B has been further implicated in osteosarcoma tumorigenesis (Brun et al., 2013) and metastasis (Muff et al., 2015). In osteosarcoma, *WNT10B* expression is regulated by the transcriptional co-factor FHL2 (four and a half LIM domains protein 2). By immunohistochemistry, FHL2 is expressed in osteosarcoma samples with increased expression in metastatic and recurrent tumors compared to normal bone. Furthermore, short hairpin knockdown of *Fhl2* in murine K7M2 osteosarcoma cells reduced β-catenin nuclear translocation and decreased the expression of *Axin2* and *Wisp-1*. sh*Fhl2* also decreased the expression of *Wnt10b* and *Wnt5a*. *In vivo,* shFHL2 reduced tumorigenesis and metastasis in mice, and sh*Fhl2* tumors had significantly less mRNA expression of *Wnt10b* and *Wnt5a* than control tumors (Brun et al., 2013).

Using microarray and array genomic hybridization, Muff et al. (2015) examined changes in gene expression between three metastatic osteosarcoma cell lines (LM5, 143B, and LM8) compared to their respective parent lines (SAOS, HOS, and Dunn). In all three metastatic lines, there was a significant upregulation of *WNT10B*, suggesting the role of WNT10B in osteosarcoma metastasis (Muff et al., 2015).

### 17.7 WNT10B and leukemia

Several studies have been conducted on the role of WNT10B in AML. The first paper showed that AC133^+^ (a glycosylation-dependent epitope of CD133 that marks stem and progenitor cells) AML cells overexpressed and released WNT10B into the microenvironment of AML patients. However, the WNT10B overexpression was not detected in normal AC133^+^ cells (Beghini et al., 2012). The same lab then used a zebrafish embryo model of AML. They found that transiently overexpressed *wnt10b* resulted in an expansion of the erythromyeloid, myeloid and erythroid progenitor cell populations, while decreasing the population of circulating neutrophils. This expansion of hematopoietic precursors at the expense of differentiated cell types, such as neutrophils, resembles pre-leukemic HSC expansion suggesting a function for *wnt10b* in leukemia generation. Subsequently, using a cohort of healthy donors, favorable AML responders, and intermediate/unfavorable responders, WNT10B was shown to be expressed in only AML patients and not in healthy donors (Lazzaroni et al., 2016). They evaluated receptors of WNT10B signaling and found that in AML, WNT10B signals through FZD4 and FZD5, whereas in T-ALL, WNT10B signals through FZD6 (Lazzaroni et al., 2016; Cassaro et al., 2021).

### 17.8 WNT10B and skin cancer

WNT10B has been implicated as a driver of normal skin development [see Section 11 and Wend et al. (2012)]. *WNT10B* expression is upregulated in skin squamous cell carcinomas (Bhatia and Spiegelman, 2005) and older recurrent melanoma patients with tumor-positive sentinel lymph nodes (Menefee et al., 2020). In addition, *Wnt10b* is upregulated in mouse tumor models of melanoma (Lu H. J. et al., 2018).

Adenoviral expression of *Wnt10b* in normal skin keratinocytes led to cellular transformation, increased proliferation, and increased motility. Furthermore, WNT10B increased the expression of genes in the WNT, EGF, and MAPK pathways. These events could all be blocked by DKK1 (Lei et al., 2015).

In contrast to an oncogenic role for WNT10B in melanoma described previously, in a mouse cell line model of melanoma (B16F10), WNT10B reduced melanoma cell proliferation, induced cellular senescence, and enhanced tyrosinase (a marker of differentiation) activity *in vitro*. Furthermore, the injection of WNT10B protein into the tumor (subcutaneous injection of B16F10 melanoma cells) in mice inhibited tumor growth (Misu et al., 2015). These conflicting results present more questions that should be addressed on whether WNT10B in melanoma patients is beneficial, harmful, or both.

### 17.9 WNT10B and colorectal cancers

Almost all colorectal cancers (92%) have mutations and/or activation of the WNT pathway, notably mutations in APC (40%–80%) or β-catenin (5%–6%) (Schatoff et al., 2017). However, the role of WNT10B in colon adenocarcinoma is not well documented. A bioinformatics study in patients with colon adenocarcinoma demonstrated that high *WNT10B* expression was an independent predictor of worse overall survival in patients (Ruan et al., 2020). Low expression of miR-148a (Table 1), which targets *WNT10B*, was also correlated with worse overall survival, metastasis, and TNM stage, suggesting a role for WNT10B in prognosis as well (Shi et al., 2019). Cisplatin-resistant, stem-like SW480 cells had a decrease in miR-148a and an increase in *WNT10B* expression. Although a miR-148a mimic decreased sphere formation, invasion, and migration, *WNT10B* overexpression increased sphere formation, invasion, and migration (Shi et al., 2019).

Interestingly, in the CaCo-2 colon cancer cell line, filtrates from the blue–green microalga *Spirulina platensis* significantly decreased the autocrine secretion of WNT10B and had anti-proliferative and pro-apoptotic effects (Smieszek et al., 2017). However, the mechanism of WNT10B suppression was not elucidated.

Inositol hexaphosphate (IP6) and its molecular skeleton, inositol (INS), are natural compounds found in grains and legumes and have antitumor effects independently and in combination with each other. Liu et al. (2020e) evaluated gene expression changes in CRC liver metastasis *in vivo* in response to treatment with IP6, INS, or a combination of both (IP6 + INS) using the mouse CT26 colorectal cell line, orthotopically. They revealed through RNA-sequencing, RT-qPCR, and western blotting that the combinatorial treatment of IP6 + INS caused a decrease in the expression of WNT10B, TCF7, and c-MYC. They also found a decrease in rates of metastasis in the IP6 + INS group and proposed that this natural drug treatment inhibited metastasis by inhibiting WNT10B signaling. However, more studies should be conducted to make this conclusion (Liu et al., 2020e).

### 17.10 WNT10B and gastric cancer

WNT10B is overexpressed in gastric cancer compared to paired normal tissue and correlates with a higher rate of lymph node metastasis. Using shRNA-mediated *WNT10B* knockdown in SGC-7901 gastric cancer cells, Wu X. D. et al. (2017) showed that *WNT10B* knockdown inhibited gastric cancer proliferation and migration *in vitro*. Additionally, sh*WNT10B* cells showed a switch from mesenchymal to epithelial with downregulation of N-cadherin and upregulation of E-cadherin in SGC-7901 cells. They also showed a positive correlation between WNT10B and cancer stem cell markers OCT4 and NANOG in gastric cancer tissues (Wu X. D. et al., 2017). This study provides a solid basis for further study on the mechanisms and potential therapeutic targets of WNT10B in gastric cancer.

### 17.11 WNT10B and brain cancer

A bioinformatics analysis of all 19 WNT ligands in human gliomas revealed an overexpression of *WNT5A* and a decreased expression of *WNT10B* in tumors compared to normal tissue. Furthermore, the overexpression of *WNT5A* and *WNT16* correlated with worse overall survival in glioma patients, whereas high expression of *WNT5B*, *WNT10B*, and *WNT3* correlated with better overall survival probability. In addition, *WNT5A* was positively correlated and *WNT10B* inversely correlated with glioma grade (Xu et al., 2020).

Conversely, Li et al. (2018) suggested that WNT10B is a driver of tumor progression in glioblastoma cells. They found that *WNT10B* and fatty acid-binding protein 4 (*FABP4*) were upregulated by RT-qPCR in malignant gliomas *versus* normal tissue. In glioma-derived U-87MG cells, sh*FABP4* significantly reduced the expression of *WNT10B* by immunofluorescence and RT-qPCR. Additionally, short hairpin knockdown of *WNT10B* reduced wound healing and invasion compared to control U-87MG cells. *FABP4*-silenced cells treated with constitutively active WNT10B rescued the reduced migration and invasion of the *FABP4*-silenced cells alone (Li H. Y. et al., 2018). Future studies will be needed to better understand the mechanistic role of WNT10B to determine if WNT10B is an oncogene or tumor suppressor in brain cancer.

### 17.12 WNT10B and endometrial cancer

*WNT10B* is significantly upregulated in endometrial cancerous tissues and cell lines compared to normal tissue (Chen et al., 2013; Zhou et al., 2016; Liu et al., 2020a). By immunohistochemistry of the proliferative phase, secretory phase, simple hyperplasia, complex hyperplasia, atypical hyperplasia, and endometrial carcinoma tissues, WNT10B had increased expression in the cancerous endometrial carcinoma samples. Furthermore, high expression of WNT10B was correlated with endometroid-type tumors, lower metastasis, and better overall survival (Chen et al., 2013). In comparison, *in vitro* cell studies show that the proliferation of Ishikawa 3-H-12 and AN3CA endometrial carcinoma cell lines was higher when *WNT10B* was overexpressed relative to control. This finding was confirmed using si*WNT10B* transfection. Additionally, *WNT10B* knockdown increased cell apoptosis rates compared to the control groups (Chen et al., 2013; Zhou et al., 2016).

In endometrial cancer stem cells, WNT10B activity is regulated by the matricellular glycoprotein SPARC-related modular calcium binding 2 (SMOC-2). SMOC-2 activates WNT/β-catenin signaling and cannot directly interact with WNT3A or WNT10B. Instead, SMOC-2 can enhance the binding between FZD6 and WNTs −3A and −10B or the binding between LRP6 and WNTs −3A or −10B. In the cancer stem cells, as assessed by proximal ligation assay, SMOC-2 can bind in a trimer with FZD6 and LRP6, and this trimer increases chemoresistance to paclitaxel and cisplatin (Lu et al., 2019).

Two studies have shown post-transcriptional regulation of *WNT10B* in endometrial cancer. One group found that miR-148a (Table 1) is significantly downregulated in cancer-associated fibroblasts in endometrial carcinoma patients and that WNT10B was upregulated. They showed through TargetScan predictions and luciferase assays that miR-148a binds directly to *WNT10B* mRNA to inhibit its expression. Furthermore, they demonstrated that WNT10B is directly responsible for the increase in tumor cell motility in endometrial cancer cells (Aprelikova et al., 2013).

Second, a positive association between WNT10B and the lncRNA HOXB-AS1 was found, along with a negative correlation between WNT10B and miR-149-3p (Table 1). miR-149-3p binds to the 3’-UTR of *WNT10B* to suppress expression, whereas HOXB-AS1 sponges miR-149-3p to allow for expression of *WNT10B.* Furthermore, HOXB-AS1 increased proliferation, migration, and invasion, whereas the addition of miR-149-3p mimics reduced this phenotype (Liu et al., 2020a).

### 17.13 WNT10B and prostate cancer

Madueke et al. (2019) compared the temporal and spatial expression of *Wnt10b* in normal and cancerous rat prostate development and metastasis. They found that when *Wnt10b* is expressed in early development, it slows growth. This reduced expression of *Wnt10b* is essential for branching morphogenesis. Then, they examined the Probasin/TAg prostate cancer rat model and found that prostate cancer had a significantly decreased expression of *Wnt10b* compared to normal prostate tissue. However, the metastatic prostate cancer cell lines HuSLC and PC3 had higher *WNT10B* expression than normal prostate tissue, and the very aggressive cell line PC3M31 had even higher *WNT10B* expression, significantly more so than PC3 cells, demonstrating different effects of WNT10B, depending on the timing of expression. Metastatic castrate-resistant patient samples also had higher *WNT10B* expression than localized prostate cancer. In PC3 cells, *WNT10B* knockdown produced significantly higher proliferation and migration rates. Furthermore, *in vivo, WNT10B* knockdown in PC3 cells led to significantly larger tumors than in the PC3 control cells. However, the *WNT10B* knockdown cells completely lost their metastatic capability. *WNT10B* knockdown cells resulted in a mesenchymal to epithelial switch. They also found a reduction in the stem cell population in *WNT10B* knockdown cells by FACS analysis and a reduction in SOX2 and NANOG expression (Madueke et al., 2019). To support this conclusion, Hu et al. (2017) found that *WNT10B* expression was localized in normal prostate spheroids that were quiescent and stem-like (Hu et al., 2017).

The endocrine-disrupting chemicals 17β-estradiol-3-benzoate (EB) and bisphenol A (BPA), which increase susceptibility to prostate cancer, decreased the DNA methylation of *Wnt10b* and subsequently increased the expression of *Wnt10b* in young rats. The combined expression of *Wnt10b* and six other methylated genes is associated with shorter recurrence-free survival in human patients (Cheong et al., 2016).

In contrast to the study by Madueke et al. (2019), in PC3 cells, *WNT10B* knockdown in the human prostate stromal cell line 19I *in vivo* decreased tumor formation and weight. Furthermore, they analyzed changes in stemness genes between control and *WNT10B* knockdown groups and found the mesenchymal stem cell markers *OCT4*, *LIF*, and *CD90* downregulated in *WNT10B-*knockdown 19I cells (Dakhova et al., 2014). Overall, WNT10B’s mechanism in prostate cancer, particularly in cancer stem cell population, requires further study.

### 17.14 WNT10B and liver cancer

In hepatocellular carcinoma (HCC) specimens, *WNT10B* is significantly upregulated compared to normal liver tissue (Zhang J. et al., 2021). In HepG2 cells, *WNT10B* shRNA knockdown significantly reduced proliferation and migration. Furthermore, *WNT10B* knockdown resulted in an increased rate of apoptosis and a slowing of the cell cycle, with more cells present in the G0–G1 phase and fewer cells in the S phase compared to control HepG2 cells (Wu et al., 2015).

Overexpression of CORO6, an actin-binding protein and a possible driver of HCC progression, upregulated *WNT10B* by RT-qPCR. CORO6 overexpression also enhanced WNT signaling activation in TOPFLASH reporter assays compared to control cells. *In vivo*, Hep3B HCC cells with shRNA knockdown of *CORO6* significantly decreased the growth rate of tumors. Protein levels of WNT10B, c-MYC, AXIN2, and cyclin D1 were downregulated in the *CORO6-*depleted tumors compared to the control Hep3B tumors (Zhang J. et al., 2021).

*WNT10B* has been identified as the target of several lncRNAs and miRNAs in HCC progression. Zhou et al. (2020) investigated lncRNAs and miRNAs in HCC and found that LINC003355:8 enhanced cell proliferation, migration, and invasion, whereas miR-6777-3p reduced proliferation, migration, and invasion. They identified *WNT10B* as a target of miR-6777-3p and that LINC00355:8 activates WNT10B by sponging miR-6777-3p to increase the expression of WNT signaling proteins β-catenin and c-MYC and inducing epithelial-to-mesenchymal transition markers (Table 1) (Zhou et al., 2020).

Two papers by Zhang et al. investigated the roles of WNT10B in HCC progression. First, while studying the effects of the transcriptional regulator NSD1 on HCC progression, they found a significantly positive correlation between *NSD1* and *WNT10B* in HCC cells. CRISPR-mediated knockout of *NSD1* since its gene KO enhanced the closed chromatin mark H3K27me3 in the promoter region of *WNT10B*, thereby reducing the expression of *WNT10B*. They also found that *NSD1* knockout reduced WNT/β-catenin signaling in cells and observed a reduction in protein expression of β-catenin, c-MYC, and cyclin D1. They found that silencing *WNT10B* in conjunction with *NSD1* further reduced WNT/β-catenin signaling as well as proliferation, migration, and invasion, whereas knocking down *WNT10B* in cells overexpressing *NSD1* neutralized the effects. *In vivo*, *WNT10B* knockdown and *NSD1* knockout were sufficient to reduce tumor volume and weight, and the combination knockdown/knockout further suppressed tumor volume and weight and reduced pulmonary metastasis significantly (Zhang et al., 2019). Using The Cancer Genome Atlas Liver Hepatocellular Carcinoma dataset, Zhang S. et al. (2021) identified the lncRNA KB-68A7.1, which inversely correlated with HCC prognosis. In their model system, they found that expression of KB-68A7.1 reduced the demethylation of histone 3 at lysine 36 (H3K36me2) and increased histone 3 lysine 27 trimethylation (H3K27me3) at the *WNT10B* promoter to decrease *WNT10B* expression. Additionally, overexpression of KB-68A7.1 sequestered NSD1 to the cytoplasm (Table 1). Consistent with this result, overexpression of KB-68A7.1 reduced the protein expression of WNT10B in HCC and hepatoma cell lines SNU-398 and HuH-7, and those with KB-68A7.1 knockdown increased expression of WNT10B in SNU-398, SK-HEP1, and THLE-3 cells. Furthermore, overexpression of KB-68A7.1 reduced proliferation, migration, invasion, and increased apoptosis rates, whereas WNT10B overexpression attenuated these effects. These results suggest a mechanism in which KB-68A7.1 functions as a tumor suppressor by sequestering NSD1 to the cytoplasm and inhibiting the transcription of *WNT10B* (Zhang S. et al., 2021)*.*

Another lncRNA, CTB-193M12.5, is overexpressed in HCC and correlates with aggressiveness and poor overall survival. CTB-193M12.5 bound to NSD1 in RNA-protein pull-down assays and enhanced the binding of NSD1 to the *WNT10B* promoter for increased transcription of *WNT10B* (Table 1)*.* Knockout of CTB-193M12.5 decreased proliferation, apoptosis, migration, and invasion, whereas overexpression of WNT10B reversed these effects (Zhang et al., 2022). In summary, NSD1 increased the transcription of *WNT10B* in HCC, and upstream lncRNAs modulated this interaction by inhibiting (KB-68A7.1) or activating (CTB-193M12.5) NSD1 and WNT10B, thereby contributing to HCC progression.

### 17.15 WNT10B and cholangiocarcinoma

In the first study of WNT10B in cholangiocarcinoma (biliary tract cancer), WNT10B was inhibited by miR-370 (Table 1), which in turn is inhibited by IL6. While the function of WNT10B in cholangiocarcinoma was not described, miR-370 overexpression reduced proliferation and was downregulated in cholangiocarcinoma *versus* normal tissue (An et al., 2012). Therefore, although the mechanism of WNT10B in cholangiocarcinoma is unknown, WNT10B reduction appears to correlate with less tumor proliferation.

## 18 Additional *WNT10B* polymorphisms

Many *WNT10B* polymorphisms have been identified in various conditions across species. Thus far, we have discussed polymorphisms in the *WNT10B* locus associated with obesity, dental anomalies, SFHM, and bone mineral density in humans. Additionally, the G607C polymorphism in the *WNT10B* promoter is associated with the Yin Deficiency pattern (a type of stroke in traditional Korean medicine that results from a deficiency of yin fluid and essence, incapable of restraining yang) and a phenomenon called bi-sup (a kind of body shape in Korean medicine) in Korean cerebral infarction patients (Ko et al., 2012; Ko et al., 2015). In cows, *WNT10B* polymorphisms in different breeds associate with body height, body length, body weight, and chest circumference (Zhao et al., 2012). In pigs, a polymorphism in the promoter of *WNT10B* is associated with litter size, as do two other WNT pathway genes [*TCF12* and catenin alpha-like protein 1 (*CTNNAL1*)] (Tao et al., 2013). In Bernese mountain dogs, an SNP near *WNT10B* is associated with elbow dysplasia (Pfahler and Distl, 2012).

## 19 Conclusion

**FIGURE 2:**
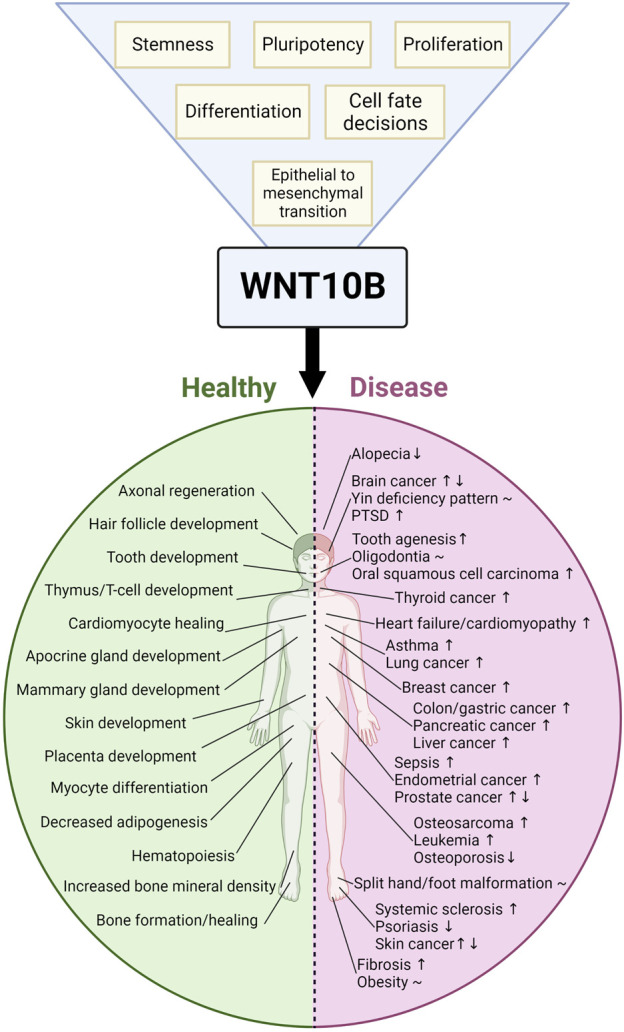
Summarizing the role of WNT10B in physiology and disease. The blue triangle with yellow squares represents across-tissue phenotypic observations of WNT10B’s function in physiology and disease, whereas the pink/green circle represents the tissue-specific roles of WNT10B. The green side represents tissues that are affected by WNT10B in a healthy developmental model, whereas the pink side represents the role of WNT10B in diseases. To the right of the disease is a symbol representing overexpression (↑), decreased expression (↓), or both (↑↓) of WNT10B. Additionally, diseases characterized by polymorphisms or mutations in WNT10B are given a (∼) symbol. Created with BioRender.com.

The knowledge and understanding of WNT10B have been greatly enhanced in the last decade. Interestingly, WNT10B has been studied not only in human, rodent, and zebrafish model systems, but also in goats, rabbits, sheep, cows, pigs, donkeys, and dogs. WNT10B is an oncogene in over 15 cancer types. WNT10B plays a role in cancer and has been implicated in the pathogenesis of osteoporosis, obesity, oligodontia, tooth agenesis, SHFM, fibrosis, PTSD, asthma, and rheumatoid arthritis (Figure 2).

WNT ligands regulate stem cell biology, and WNT10B has been ascribed roles in mesenchymal, mammary, prostate, skin, hematopoietic, dental pulp, and cancer stem cells. WNT10B expression correlates with stem cell proliferation or differentiation, but the mechanism of action of WNT10B in stem cells is not well defined. This is an important area for future studies in regenerative medicine and anti-cancer therapies.

Although several WNT-signaling inhibitors are in early clinical trials for various cancer types, no WNT-specific drugs have been FDA-approved for cancer to date (Harb et al., 2019). In contrast, the WNT signaling activator (the anti-sclerostin antibody EVENITY) is approved for osteoporosis. However, this may not be through the activation of WNT10B signaling, as there remains an increase in bone mineral density in the WNT10B knockout mice treated with the anti-sclerostin antibody. Nevertheless, targeting WNT10B signaling could lead to therapies for many conditions.

The past decade has focused on *WNT10B* regulation. Numerous miRNAs, lncRNAs, and a circRNA regulate *WNT10B* (Table 1). miR-148a, the most studied of the RNAs, regulates *WNT10B* in adipocytes, lung fibrosis, pancreatic cancer, oral squamous cell carcinoma, colon adenocarcinoma, endometrial carcinoma, and thyroid cancer. In addition, many transcription factors and chemicals (such as butyrate from probiotics) regulate *WNT10B*.

In addition to therapeutics and understanding more about WNT10B regulation in different cell types, emerging areas for WNT10B studies may include fetal growth restriction and placenta biology, immune-oncology, and the tumor microenvironment in various cancer types. The work in the last decade on understanding the role of WNT10B in a variety of normal and disease processes is fascinating and holds exciting promise for enhancing our future understanding of normal development, pathogenesis, and treatment.
